# Spectroscopic and Theoretical Studies of Ruthenium Complexes with a Noninnocent N_2_S_2_ Ligand in Different Redox States

**DOI:** 10.1021/acs.inorgchem.5c02059

**Published:** 2025-07-26

**Authors:** Javier A. Luna, Kyle D. Spielvogel, Nathan R. Loutsch, Sydney M. Loria, Leah P. Weisburn, Mark R. Ringenberg, Bess Vlaisavljevich, Jason M. Keith, Scott K. Shaw, Scott R. Daly

**Affiliations:** † Department of Chemistry, 4083 The University of Iowa, E331 Chemistry Building, Iowa City, Iowa 52242, United States; ‡ Department of Chemistry, 3719 Colgate University, 13 Oak Drive, Hamilton, New York 13346, United States; § Universität Stuttgart, Institut für Anorganische Chemie, Pfaffenwaldring 55, Stuttgart 70569, Germany

## Abstract

Herein we report an electronic structure investigation of neutral and oxidized Ru complexes containing a redox noninnocent N_2_S_2_ ligand derived from *o*-phenylenediamide (**L1**). UV–vis spectroelectrochemistry (SEC) studies were conducted on the square pyramidal complex [Ru^II^(**L1**)­(PPh_3_)] (**1**) and the six-coordinate complexes [Ru^II^(μ-BH_3_)­(**L1**)­(PPh_3_)] (**2**) – which has BH_3_ bound in a metal–ligand cooperative (MLC) fashion across Ru and **L1** – and [Ru^II^(**L1**)­(PPh_3_)­(MeCN)] (**3**). The SEC results yielded spectra assigned to singly and doubly oxidized **1** and **3**, revealing electronic structure changes as a function of oxidation state and in response to the presence and absence of bound MeCN. By contrast, the SEC results of **2** showed that it rapidly loses MLC-bound BH_3_ upon oxidation. The SEC results for **1** and **3** were compared to single-crystal XRD data and UV–vis, EPR, and P K-edge, S K-edge, and Ru L_3_-edge X-ray absorption spectroscopy (XAS) data collected on isolated samples of chemically oxidized **3**. The data revealed that the first two oxidations are primarily localized on the ligand, which was supported by DFT and TDDFT calculations. DFT calculations for the doubly oxidized species revealed a singlet ground state with a singlet–triplet gap of 8.9 kcal/mol. CASPT2 calculations corroborated the DFT calculations and further revealed that the singlet ground state is multiconfigurational with 21% radical character. Collectively, the results establish redox formalisms and the underlying electronic structure of Ru complexes containing a noninnocent tetradentate ligand in different oxidation states.

## Graphical abstract

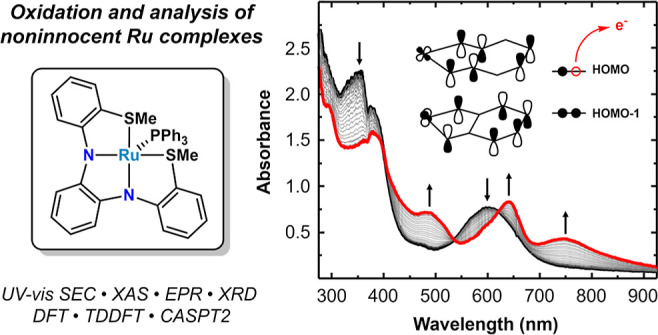

## Introduction

Noninnocent ligands are redox-active ligands that form metal complexes with ambiguous oxidation states.^1–8^ This ambiguity is often observed when there is significant mixing between metal d-orbitals and π-conjugated orbitals localized on the ligand that blur the line between formal metal and ligand redox state assignments.^9^ Among the many examples of this phenomenon are ruthenium complexes containing 1,2-disubstituted phenylene ligands like *o*-phenylenediamine, which can undergo successive one-electron oxidations to form the *o*-benzosemiquinonediimine radical followed by the fully oxidized *o*-benzoquinonediimine.^10–14^ Extensive metal–ligand covalency between phenylene π orbitals and π-type Ru 4d orbitals (as well as those for other transition metals) often yields diffuse molecular orbitals and electronic structures that can be highly sensitive to local structure, ligand substituents, redox states, and solvent effects. In this context, an important goal is to understand how these factors influence the properties and reactivity of metal complexes containing redox noninnocent ligands, especially in cases where the ligands themselves can participate in chemical reactions and promote catalysis.^15–22^

**Scheme 1:**
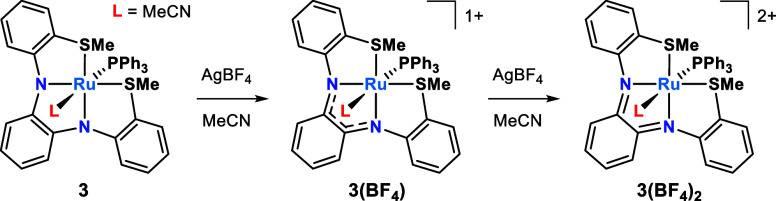
Chemical Oxidation Studies of **3** in MeCN

We previously reported a series of Ru complexes containing a redox-active N_2_S_2_ ligand derived from *o*-phenylenediamide (**L1**; Figure 1a).^23–25^ Among these was [Ru­(**L1**)­(PPh_3_)] (**1**; Figure 1b), a square pyramidal complex that undergoes two reversible one-electron oxidations consistent with the ligand-centered redox activity expected of the *o*-phenylenediamide subunit (Figure 1a). The open coordination site trans to PPh_3_ in **1** is capable of binding ancillary ligands depending on the field strength. For example, **1** does not appear to bind weaker field ligands like neutral etherates such as thf due to the strong trans influence of PPh_3_, but it will coordinate stronger field ligands such as good σ donors/weak π-acceptors like MeCN to form [Ru­(**L1**)­(MeCN)­(PPh_3_)] (**3**; Figure 1b). Moreover, we showed how **1** readily undergoes metal–ligand cooperative (MLC) binding^26^ with BH_3_ to form [Ru­(μ-BH_3_)­(**L1**)­(PPh_3_)] (**2**), as shown in Figure 1b.^23,24^

**Figure 1:**
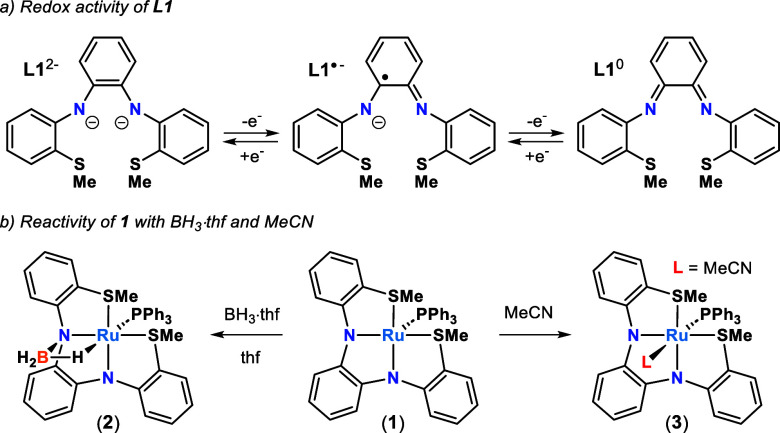
(a) Select resonance structures of **L1** in different redox states. (b) Structures of **1**–**3**.

As is typical of Ru complexes containing redox active ligands like **L1**, it was not clear in our initial studies of **1**–**3** as to how to assign the formal metal and ligand oxidation states as the complexes were oxidized,^23^ especially given the accessible higher oxidation states for Ru. In this context, we have shown that the locus of oxidation in square-planar Ni complexes containing **L1** can be quite sensitive to the coordination of ancillary ligands to the metal. The first oxidation of [Ni^II^(**L1**)] was localized on the ligand to form [Ni^II^(**L1** ^•^)] with a ligand centered radical, but subsequent coordination of pyridine in an axial coordination site induced a valence tautomerism to form [Ni^III^(**L1**)­(py)] with the oxidation localized on the metal.^27^

Here we expand upon prior electrochemical studies of **1**–**3** to investigate how occupation of the open coordination site in **1** with MeCN and MLC-bound BH_3_ affects the locus of oxidation with **L1** and Ru. Because some of the complexes cannot be isolated due to their instability beyond the electrochemical time scale, we have employed spectroelectrochemical (SEC) studies to capture redox speciation and electronic structure at different electrochemical potentials. These spectra collected in situ are compared to structural and spectroscopic data (UV–vis, XAS, EPR spectroscopy) obtained on structurally authenticated samples of **3** in different redox states. Theoretical calculations reproduce the experimental observations, and we discuss how the data converge to show that the first two oxidations in these Ru complexes are primarily centered on the ligand.

## Results

To provide a foundation for spectroelectrochemical and chemical oxidation studies described in the following sections, we will start with the electronic structure analysis of the isolated parent complexes **1**, **2**, and **3**. The UV–vis spectra of **1** and **2** in thf and **3** in MeCN are shown in Figure 2. The spectrum of five-coordinate **1** revealed three prominent and relatively intense absorptions at 264, 354, and 597 nm (37,878, 28,248, and 16,750 cm^–1^; Table 1). The lowest energy feature observed for **1** at 597 nm disappears in the spectra **2** and **3**, and the broad absorption at 354 nm in **1** gives way to two lower intensity features at 331 and 412 nm (30,211 and 24,271 cm^–1^) for **2** and 359 and 407 nm (27,855 and 24,570 cm^–1^) for **3**. In general, **2** and **3** have a similar spectral profile despite the presence of MLC in **2** and the differences in solvent (thf and MeCN), but the spectrum of **2** is blue-shifted with respect to **3**.

**Figure 2:**
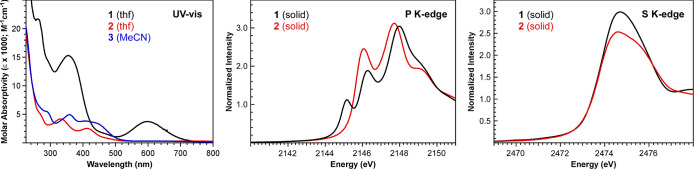
Left–UV–vis spectra collected on **1** and **2** in thf and **3** in MeCN. The spectrum of **2** was collected in the presence of excess BH_3_·thf. Middle and right–Normalized P and S K-edge XAS spectra collected on solid samples of **1** and **2**.

**Table 1:** UV–Vis Data Collected on Neutral and Oxidized Samples of 1–3

UV–vis data collected on isolated complexes				UV–vis spectroelectrochemistry data				
complex (solvent)	λ (nm)	energy (cm^–1^)	ε (M^–1^ cm^–1^)	solvent	species	λ (nm)	energy (cm^–1^)	ε^a^ (M^–1^ cm^–1^)
**1**	597	16,750	4460	thf	**1**	600	16,667	4.5 × 10^3^
(thf)	354	28,248	13,900			375	26,667	1.1 × 10^4^
	264	37,878	21,600			355	28,169	1.3 × 10^4^
					**1** ^**+**^	750	13,333	2.5 × 10^3^
**1**	601	16,639	3700			640	15,625	4.9 × 10^3^
(CH_2_Cl_2_)	356	28,090	15,200			485	20,619	4.2 × 10^3^
	264	37,878	21,600			375	26,667	1.1 × 10^4^
						295	33,898	1.1 × 10^4^
**2**	412	24,271	3150		**1** ^**2+**^	560	17,857	9.7 × 10^3^
(thf)	331	30,211	1840			425	23,529	6.0 × 10^3^
	270	37,037	4920					
				thf	**2**	410	24,390	4.3 × 10^3^
**3**	407	24,570	3880			330	30,303	7.1 × 10^3^
(MeCN)	359	27,855	4800		**2** ^**+**^	525	19,048	5.2 × 10^2^
	286	34,965	4540			360	27,778	1.4 × 10^3^
						315	31,746	2.0 × 10^3^
**3(BF**_**4**_ **)**	707	14,145	2600					
(CH_2_Cl_2_)	602	16,606	4500	MeCN	**3**	420	23,810	2.7 × 10^3^
	510	19,625	5200			360	27,778	3.5 × 10^3^
	479	20,879	5400			290	34,483	3.5 × 10^3^
	393	25,434	10,500		**3** ^**+**^	710	14,085	5.9 × 10^2^
	376	26,569	11,200			605	16,529	9.8 × 10^2^
	293	34,116	13,700			510	19,608	1.2 × 10^3^
						375	26,667	2.6 × 10^3^
**3(BF**_**4**_ **)**_**2**_	546	18,312	14,400		**3** ^**2+**^	540	18,519	2.6 × 10^3^
(CH_2_Cl_2_)	428	23,341	8700			410	24,390	1.4 × 10^3^
	340	29,372	7200					
	263	37,990	23,500					

a Based on starting analyte concentration and assuming 100% conversion during electrolysis.

Ligand K-edge X-ray absorption spectroscopy (XAS) data were collected on solid samples of **1** and **2** at the P and S ligand K-edges to complement the UV–vis analysis (Figure 2). Ligand K-edge XAS, where ligand refers to the type of atom bound to the metal (in this case P and S), uses synchrotron-generated X-rays to probe dipole-allowed transitions from ligand 1s orbitals into antibonding MOs containing ligand p character.^28–32^ These absorptions are typically observed in the pre-edge region of the XAS spectrum, and their transition intensity is dependent on the amount of ligand p character mixing in the antibonding wavefunction. This makes ligand K-edge XAS a powerful technique for experimentally quantifying metal–ligand covalency in coordination complexes. As shown here, it can also be used to interrogate the energy and orbital composition of unoccupied molecular orbitals. These data fall in a similar energy window in the “tender” X-ray region, and they do not require ultrahigh vacuum (UHV) conditions like lower-energy N K-edge data (which were not collected). Samples of **3** could not be analyzed by XAS because they readily lose coordinated MeCN when isolated from the mother liquor, as described previously,^23^ but XAS analysis of oxidized samples of **3** with more strongly bound MeCN will be discussed below.

The P K-edge XAS spectra of **1** and **2** yielded pronounced differences consistent with the changes observed by UV–vis spectroscopy. The P K-edge XAS spectrum of **1** revealed a resolved pre-edge feature at 2145.1 eV, and two higher energy features at 2146.2 and 2147.9 eV (Table 2). Interestingly, the lowest energy pre-edge feature at 2145.1 eV disappears in the spectrum of **2**. Only the two higher energy features are observed at 2145.9 and 2147.7 eV, but the former feature increases in intensity relative to the same feature in **1**. In contrast to the P K-edge data, S K-edge XAS spectra of **1** and **2** revealed only subtle differences in intensity and profile that were slightly more apparent when looking at the second derivative traces.

**Table 2:** X-Ray Absorption Spectroscopy Peak Positions Based on 2nd Derivative Traces

complex	P K-edge	S K-edge
**1**	2145.1	2474.3
	2146.2	2475.9
	2147.9	
**2**	2145.9	2474.3
	2147.7	2475.4
	2149.2	2475.7
**3(BF**_**4**_ **)**	2144.4	2474.0
	2146.1	2475.7
	2148.0	
	2149.4	
**3(BF**_**4**_ **)**_**2**_	2144.4	2474.1
	2146.3	2476.1
	2148.0	
	2149.3	

Density functional theory (DFT) calculations were performed to interpret the electronic spectra of **1**–**3**. The calculations were performed using dispersion-corrected B3LYP with LANL08­(f) and double-ζ 6-31G­(d,p) basis sets because we have shown that this level of theory performs consistently well for modeling experimental spectra using time-dependent density functional theory (TDDFT).^33–44^ Moreover, it has been reported by others that B3LYP provided the most accurate prediction of XAS transitions for Ru complexes in comparative studies with different DFT functionals.^45^ As shown in Table S2 (Supporting Information), the calculated bond distances and angles of the final structures in solution are in good agreement with the experimental changes observed by single-crystal XRD.

A truncated MO diagram for **1**–**3** with Kohn–Sham plots of the most relevant orbitals is provided in Figure 3. By omitting the phenyl groups on PPh_3_, the point group symmetry of **1** and **3** can be approximated as *C*_s_ with the lone reflection plane bisecting the N_2_S_2_ ligand and containing the Ru–P bond. By comparison, the bound BH_3_ group in **2** breaks the reflection plane and reduces the point group symmetry to *C*_1_. Given the low symmetry of **1**–**3**, and to help better facilitate comparisons between MOs in the *C*_s_ and *C*_1_ point groups, we use the σ and π labels shown in Figure 3 instead of Mulliken symbols in reference to the type of metal–ligand bonding interactions. The most relevant frontier MO energies and orbital contributions are provided in Table 3.

**Figure 3:**
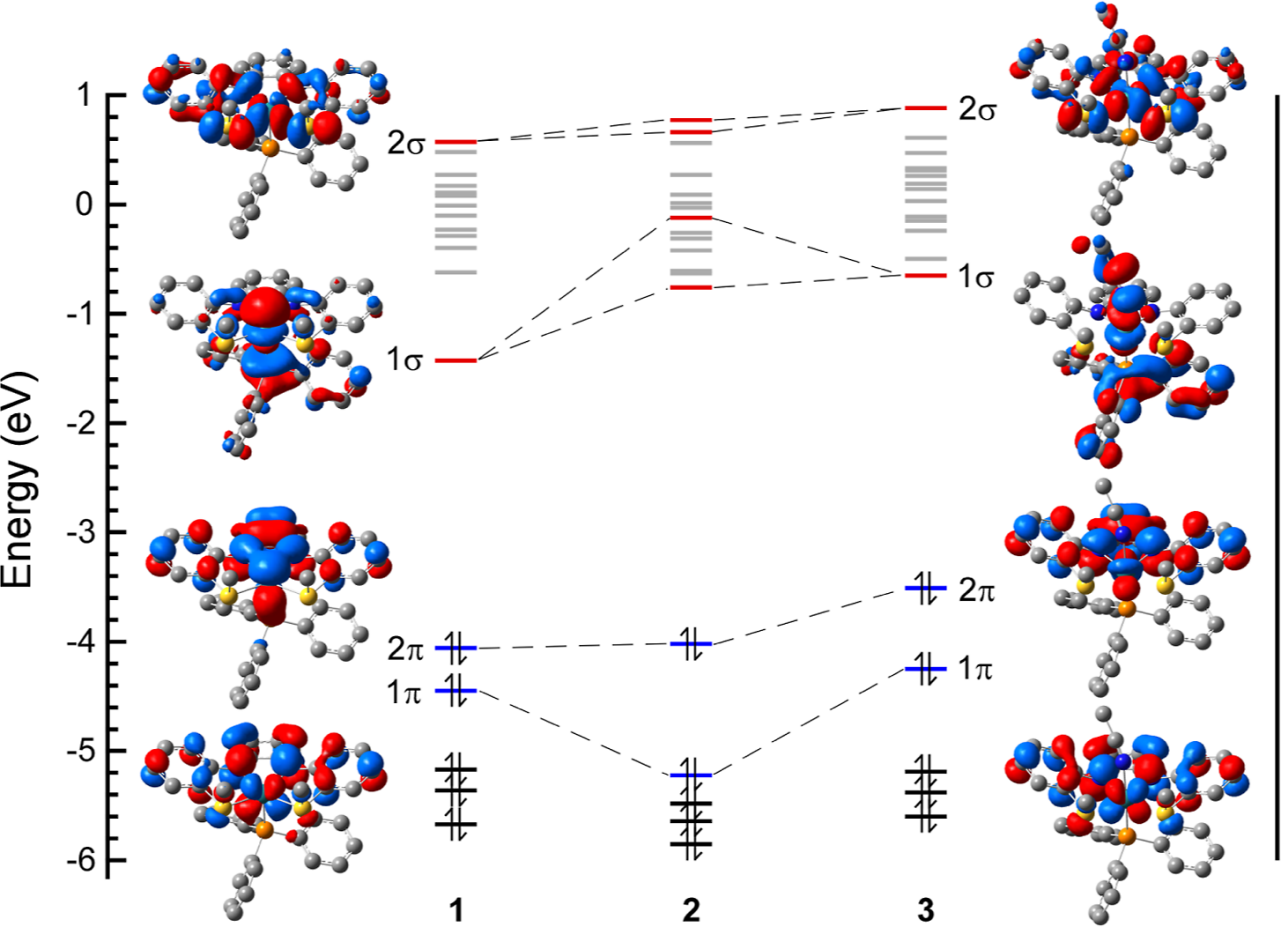
Truncated MO diagram for **1**–**3** from DFT calculations. The Kohn–Sham plots depict the metal-based MOs highlighted in blue (1π and 2π) and red (1σ and 2σ). The MO color coding matches that used for the qualitative MO diagram shown in Figure 4. Unoccupied orbitals that are primarily ligand-based in character are shown in gray.

**Table 3:** Calculated Compositions and Energies for Select Frontier MOs for 1–3^a^

complex	MO	E (eV)	Ru (4d)	N (2p)^b^	P (3p)	S (3p)	assignment
**1**	182	0.57	32%	8%		15%	d_*xy*_ (2σ)
(LUMO)	170	–1.43	33%	4%	11%	3%	d_*z* ^2^_ (1σ)
(HOMO)	169	–4.06	15%	24%	2%		d_*xz*_ (2π)
	168	–4.45	21%	25%		2%	d_*yz*_ (1π)
	167	–5.17	53%	5%	1%	12%	d_*xz*_
	166	–5.36	82%			2%	d_*x*__^2^_-y^2^
	165	–5.67	45%	4%		6%	d_*yz*_
**2**	187	0.77	14%	9%	1%	6%	d_*xy*_
	186	0.66	23%	4%	3%	12%	d_*z* ^2^_
	180	–0.12	17%	3%	1%	7%	d_*xy*_
(LUMO)	174	–0.76	11%	1%	4%	3%	d_*z* ^2^_
(HOMO)	173	–4.02	12%	29%		1%	d_*xz*_
	172	–5.22	63%	4%		14%	d_*yz*_
	171	–5.48	78%	1%		1%	d_*x*__^2^__‑*y*__^2^_
	170	–5.64	53%	6%		6%	d_*yz*_
	169	–5.85	20%	8%	1%	1%	d_*xz*_
**3**	194	0.88	24%	7%		13%	d_*xy*_ (2σ)
(LUMO)	181	–0.65	17%	1%	7%	2%	d_*z* ^2^_ (1σ)
(HOMO)	180	–3.51	10%	30%			d_*xz*_ (2π)
	179	–4.25	17%	26%		4%	d_*yz*_ (1π)
	178	–5.19	60%	2%	1%	14%	d_*xz*_
	177	–5.38	79%			1%	d_*x*__^2^__‑*y*__^2^_
	176	–5.60	54%	2%		4%	d_*yz*_

a MOs shown are those that have ≥10% Ru 4d character. Assignments are provided based on the identity of the Ru 4d orbital mixing in the MO and the labels provided in Figure 3.

b N *p*-character calculated for **L1** only. Does not include MeCN for **3**. MOs with orbital compositions less than 0.5% are shown as blanks.

Analysis of the Kohn–Sham orbitals reveals that the HOMO (2π) and HOMO–1 (1π) for **1** and **3** contain mixing between the Ru 4d_*xz*_ and 4d_*yz*_ and conjugated π-orbitals on the *o*-phenylenediamine backbone. The HOMO energy in **2** is similar to **1**, despite that the MLC-bound BH_3_ limits mixing to two of the three aryl groups in the N_2_S_2_ backbone and the Ru 4d_*yz*_. The change in conjugation has a more pronounced influence on the HOMO–1 (1π), which drops in energy due to disruption of Ru-L π bonding upon MLC binding in **2**. The LUMO for **1** (1σ) contains the Ru 4d_*z* ^2^_, which is well resolved from an envelope of higher energy ligand-centered MOs and the Ru–N/S σ* MO (2σ) derived from the Ru 4d_*xy*_. As shown in Figure 3, BH_3_ and MeCN coordination to the vacant coordination site in **1** pushes the LUMO to higher energy, indicating that the LUMO energy is governed primarily by occupation of the axial coordination site (i.e., 6-coordinate Ru in **2** and **3** vs 5-coordinate Ru in **1**). Moreover, the MLC binding in **2** causes the 1σ and 2σ to split due to loss of the *C*_s_ mirror plane (Table 3).

The origin and parentage of the highlighted orbitals in the Kohn–Sham plots can be explained using the qualitative MO diagram provided in Figure 4. Starting with only the Ru-L σ bonds and using the axis system shown in the figure, the 4d_*xy*_ and 4d_*z* ^2^_ form the Ru–N/S σ* and Ru–P σ*, respectively, with the Ru–N/S σ* being more destabilized due to the four equatorial ligand donor atoms. Introducing π bonding yields new Ru–N π and π* orbitals from mixing between the 4d_*xz*_ and 4d_*yz*_ and filled π orbitals that exist primarily on the N atoms and the *o*-phenylenediamide subunit.^46^ Notably, it is the **L1** π orbitals that are destabilized to become the HOMO and HOMO–1, as determined by comparing the calculated metal and ligand orbital contributions in the Ru–N π and π* orbitals. This Ru–N π and π* orbital parentage is inverted with respect to traditional π-donor ligands, which is attributed to the increased π conjugation afforded by the triaryl framework in **L1**.

**Figure 4:**
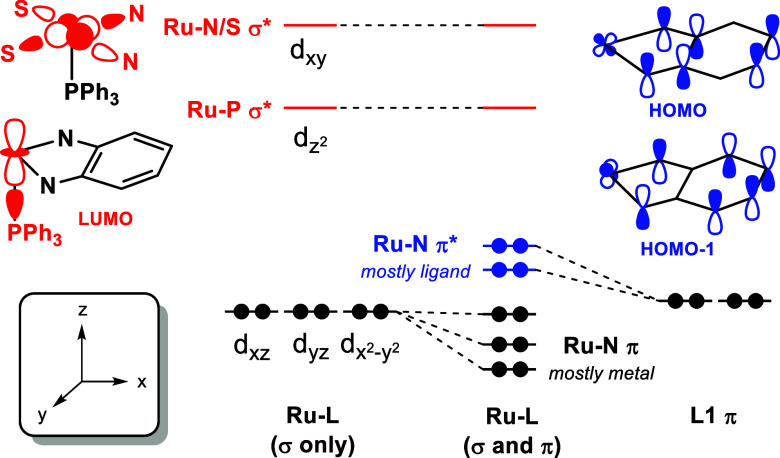
Qualitative MO diagram showing the relative splitting of the Ru 4d orbitals due to Ru-L σ bonding only and after mixing with the filled frontier π orbitals on **L1** (shown with flanking thioanisole groups omitted).

TDDFT calculations performed to simulate the spectra and assign transitions for the UV–vis and ligand K-edge XAS data are in good agreement with the experimental spectra (Figures 5 and S5). Starting with the UV–vis spectra, the unique low energy absorption observed for **1** at 597 nm (16,750 cm^–1^) can be assigned as an LMCT transition corresponding to Ru–N π* → 1σ (LUMO). These transitions are pushed to higher energy in **2** and **3**, which corresponds to the increase in LUMO energies upon axial coordination with B–H and MeCN, respectively, as shown in Figure 3. The higher energy absorption in **1** at 354 nm is comprised of a manifold of transitions, the most intense being the LMCT transition assigned as Ru–N π* → Ru–N/S σ* involving the 2π (HOMO) and 2σ shown in Figure 3. A similar manifold of transitions accounts for the higher energy absorptions for **2** and **3**. Notably, the three absorptions observed at 407, 359, and 286 nm in the spectrum of **3** contain LMCT transitions that can be assigned as 2π (HOMO) → 1σ (LUMO), 2π (HOMO) → 2σ, and 1π → 2σ, respectively, based on the primary orbital contributions associated with the calculated electronic states.

**Figure 5:**
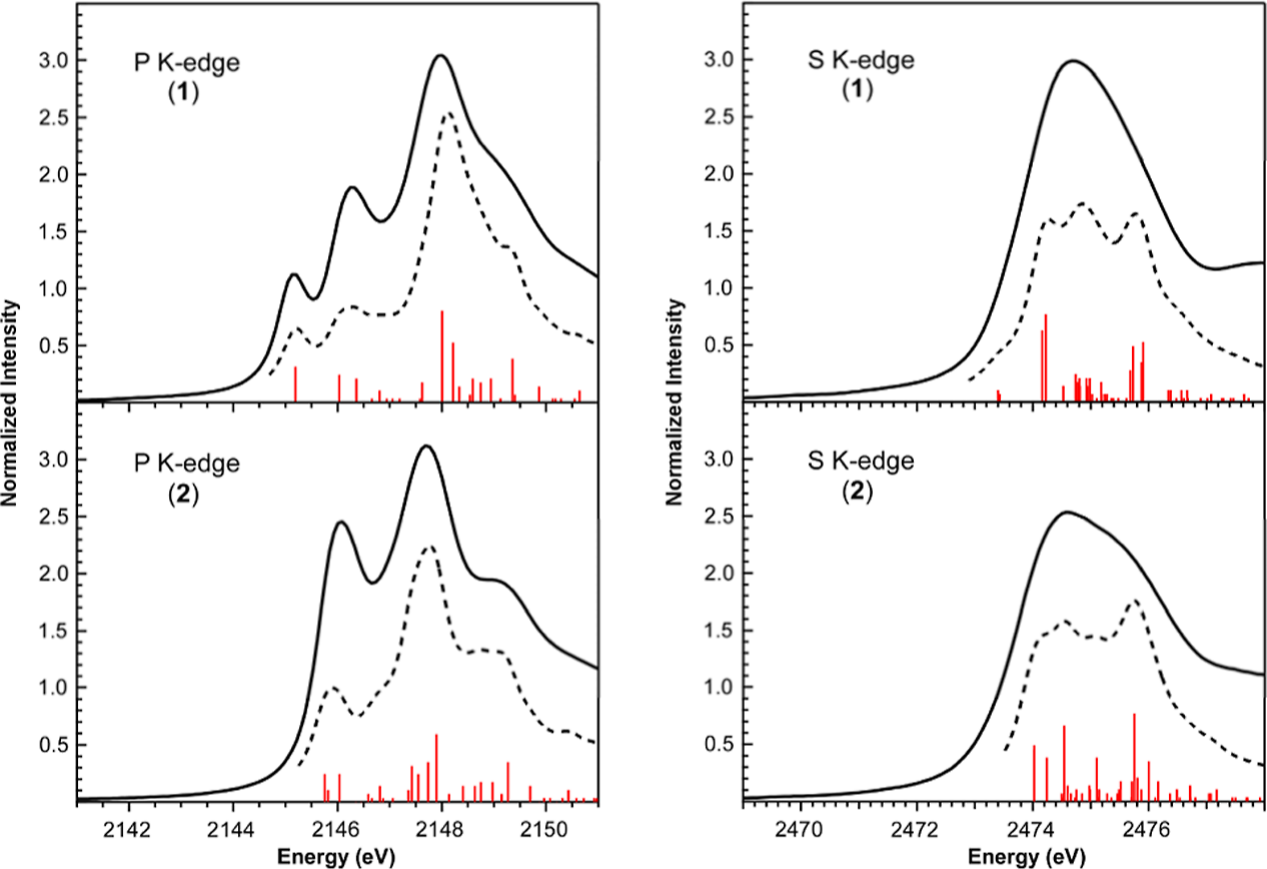
Comparison of experimental and simulated P (left) and S (right) K-edge XAS data for **1** and **2**. The experimental spectra (solid lines), simulated spectra (dashed lines), and calculated transitions (red bars) are shown. Calculated oscillator strengths were multiplied by a factor of 350 to bring them on scale with the experimental data. As described previously,^37–39,48^ an energy shift of +49.7 eV (P K) and +57.0 eV (S K) was applied to the calculated spectra so that relative differences in calculated and experimental peak positions could be compared.

TDDFT calculations conducted at the P and S K-edge XAS are also in good agreement with the experimental data and corroborate the unique spectral differences observed in the UV–vis spectra (Figure 5). The lowest energy pre-edge feature observed at 2145.1 eV in the P K-edge XAS spectrum of **1** is assigned as P 1s → Ru–P σ* (LUMO; 1σ). This transition is pushed to higher energy in the spectrum of **2** in response to MLC binding, as observed for transitions associated with the LUMO in the UV–vis spectrum. This indicates that the lowest energy feature in **2** at 2145.9 eV can be assigned to overlapping ligand and metal-based P K-edge transitions, which accounts for its increased intensity with respect to the feature at similar energy in **1**. The P K-edge XAS spectrum of **2** is similar to that reported by Grapperhaus et al. for 6-coordinate Ru­(II) complexes containing a single PPh_3_ ligand.^47^ In addition to good agreement with the P K-edge data, the TDDFT calculations account for the lack of resolution in the S K-edge XAS spectra for both **1** and **2**. Both spectra contain numerous transitions with similar energies and intensities. The subtle differences in the experimental spectral profile and normalized intensity are reflected in the simulated spectra.

### UV–vis Spectroelectrochemical (SEC) Analysis

Building on our analysis of the neutral complexes, we next evaluated how the electronic structures of **1**–**3** changed when the complexes were oxidized. One of the challenges in experimentally measuring the electronic structure of these oxidized species is their relative stability. We have only been able to chemically oxidize samples of **3** for subsequent analysis, which is likely facilitated by persistent occupation of MeCN in the coordination site trans to PPh_3_. As described in the following section, attempts to chemically oxidize **1** and **2** in thf leads to decomposition. Moreover, we have shown that **2** loses BH_3_ to form equilibrium mixtures of **1** and **2** when oxidized in cyclic voltammetry (CV) studies,^24^ which was confirmed in SEC studies described below.

To overcome the challenges isolating oxidized samples of **1** and **2**, we collected their UV–vis SEC spectra in thf so that their electronic structure could be compared to calculated electronic structure data. We previously reported that the CV of **1** in thf yields two reversible waves at −0.72 V (*I*_pc_/*I*_pa_ = 0.975) and −0.25 V (*I*_pc_/*I*_pa_ = 0.954) versus ferrocene/ferrocenium (Fc/Fc^+^, the standard for all potentials reported herein; Table 4).^23,24^ The first reversible wave at −0.72 V is assigned as the **1** ^**+**^/**1** couple and the second wave at −0.25 V is assigned as the **1** ^2+^/**1** ^+^ couple. MLC binding of BH_3_ reduces the number of reversible redox waves from two in **1** to one in **2**, which is attributed to MLC taking one of the redox active N atoms out of conjugation. For comparison, CV data were collected on **3** in MeCN prior to the SEC studies, and they are reported here for the first time (Figure 6). These data were collected in MeCN to ensure the MeCN remained bound to Ru and to evaluate its influence on the electrochemical potentials. The CV of **3** in MeCN yielded a CV like **1** in thf, but at slightly different potentials (Table 4). Two reversible waves were observed at −0.82 V (*I*_pc_/*I*_pa_ = 0.981) and −0.19 V (*I*_pc_/*I*_pa_ = 0.969). The 0.1 V shift of the **3** ^**+**^/**3** couple to more negative potentials compared to **1** ^**+**^/**1** in thf is consistent with greater ease of oxidation due to MeCN binding to the metal.

**Table 4:** Measured Redox Potentials vs. Fc/Fc^+^ for 1 and 2 in thf and 3 in MeCN with 0.1 M (^n^Bu_4_N)­PF_6_ ^a^

	*E*_1/2_ (V)	*E*_pa_ – *E*_pc_ (V)	*I*_pc_/*I*_pa_
**1** (thf)^b^	–0.72	0.16	0.975
	–0.25	0.16	0.954
**2** (thf)^a^	–0.50	0.13	0.617
**3** (MeCN)	–0.82	0.07	0.981
	–0.19	0.07	0.969

a Data collected at 100 mV/s using a glassy carbon working electrode, Pt wire counter electrode, and Pt wire quasi-reference electrode

b Reported previously in ref 24.

**Figure 6:**
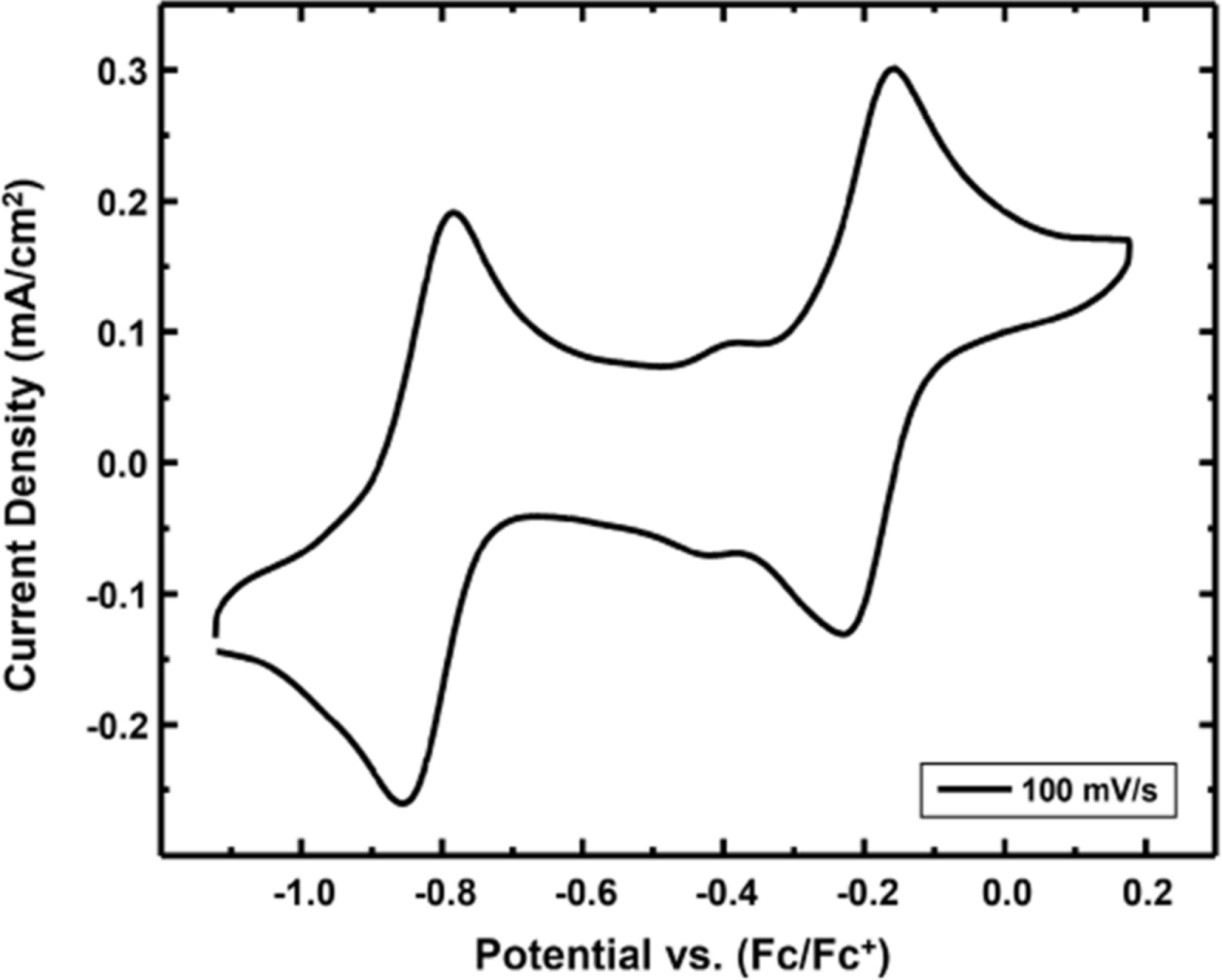
CV of **3** collected in MeCN with 0.1 M (^*n*^Bu_4_N)­PF_6_ at 100 mV/s using a glassy carbon working electrode, Pt wire counter electrode, and Pt wire quasi-reference electrode. The CV scan shows two reversible redox features at *E*_1/2_ = −0.82 V vs Fc/Fc^+^ (*I*_pc_/*I*_pa_ = 0.981), −0.19 V vs Fc/Fc^+^ (*I*_pc_/*I*_pa_ = 0.969). The feature at *E*_1/2_ = −0.40 V vs Fc/Fc^+^ is attributed to the presence of a small impurity.

UV–vis SEC analysis was performed on **1** and **2** in thf and **3** in MeCN to analyze the changes in solution as a function of potential. Using **1** as the representative example, the potential at the electrode was first held at −1.0 V vs Fc/Fc^+^ (below the first oxidation) and at each potential thereafter for 100 s before a UV–vis spectrum was collected. The potential was moved at 10 mV increments until the entire electrochemical window of the complex was scanned. The data are summarized in Table 1.

The spectra collected for **1**–**3** in the spectrochemical cell in the presence of electrolyte matched those collected on neat solutions. The starting UV–vis spectrum of **1** in the electrochemical cell is characterized by three peaks at 355, 375, and 600 nm, effectively identical to those shown in Figure 2. Oxidation of **1** to **1** ^**+**^ results in the persistence of the transition at 375 nm, disappearance of the transition at 355 nm, red shifting of the transition at 600 to 640 nm, and the emergence of a shoulder and broad peak at 485 and 750 nm (Figure 7a). Oxidation of **1** ^**+**^ to **1** ^**2+**^ results in the disappearance of transitions at 295 and 750 nm, a red shift of 375 to 425 nm and convergence of peaks 485 and 640 nm to 560 nm (Figure 7b).

**Figure 7:**
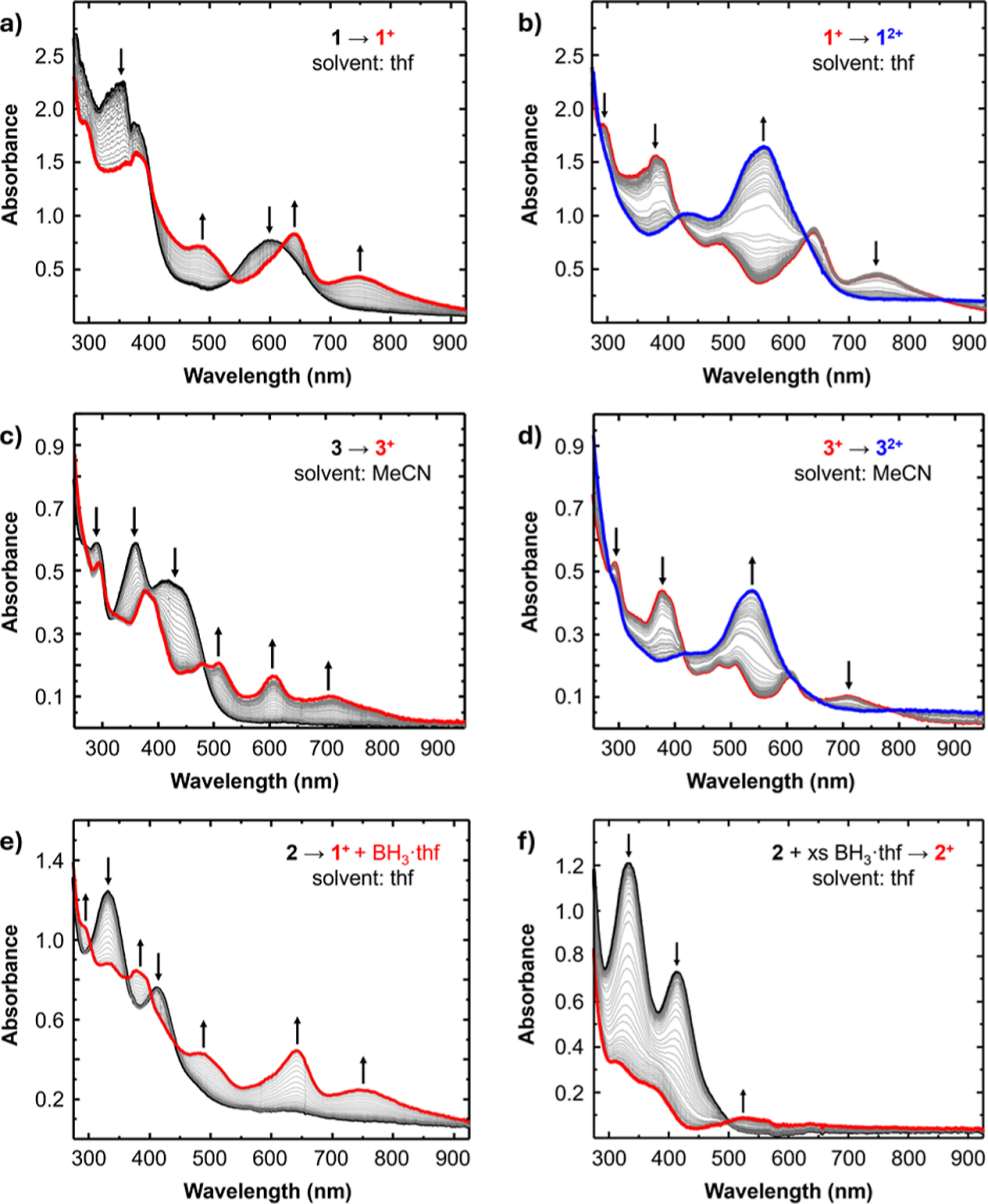
(a) UV–vis SEC spectra of **1** (black trace) oxidized to **1** ^**+**^ (red trace) in thf. Potential range: −1.0 V to −0.5 V vs Fc/Fc^+^. (b) UV–vis SEC spectra of **1** ^**+**^ (red trace) oxidized to **1** ^**2+**^ (blue trace) in thf. (c) UV–vis SEC spectra of **3** (black trace) oxidized to **3** ^**+**^ (red trace) in MeCN. Potential range: −1.2 V to −0.6 V vs Fc/Fc^+^. (d) UV–vis SEC spectra of **3** ^**+**^ (red trace) oxidized to **3** ^**2+**^ (blue trace) in MeCN. Potential range: −0.6 to 0.1 V vs Fc/Fc^+^. (e) UV–vis SEC spectra of **2** in thf (black trace) oxidized to **1** ^**+**^ (red trace). Potential range: −1.0 V to −0.1 V vs Fc/Fc^+^. (f) UV–vis SEC spectra of **2** with excess BH_3_·thf in thf (black trace) oxidized to **2** ^**+**^ (red trace). Potential range: −0.5 to 0.1 V vs Fc/Fc^+^. Experimental conditions: WE: gold honeycomb (for SEC), RE: quasi Pt, CE: Ag. Electrolyte concentration: 0.1 M (^*n*^Bu_4_N)­PF_6_. Analyte concentration: 1 mM. Cell path length: 1.7 mm.

The UV–vis SEC spectrum of **3** in MeCN is characterized by three strong absorbances at 290, 360, and 420 nm. Oxidation of **3** to **3** ^**+**^ results in a red shift of the transitions at 360 to 375 nm and 420 to 510 nm and the immergence of lower energy transitions at 605 and 710 nm (Figure 7c). Oxidation of **3** ^**+**^ to **3** ^**2+**^ results in the disappearance of the transitions at 360, 375, and 710 nm and the emergence of a broad peak at 540 nm and a small shoulder at 410 nm (Figure 7d). A broad band also appears at ca. 920 nm, which is also observed for **1** ^**2+**^ and is typical of ligand-to-ligand charge transfer (LLCT) for close-shell *o*-benzoquinonediimine ligands (see Figure S12 for an SEC spectrum capturing more of the NIR region for in situ generated **3** ^**2+**^).^49^ These spectral features for **3** ^**+**^ and **3** ^**2+**^ are consistent with those obtained for chemically oxidized samples, as described in the following section. In general, the UV–vis SEC spectra for the oxidized species of **1** and **3** reveal the same general profile with slight shifting in the energy of the absorption features.

As reported in our previous CV studies, **2** appears to lose BH_3_ upon oxidation in thf to form **1** ^**+**^. The SEC spectra collected during the oxidation of **2** spectroscopically confirms this by showing the ingrowth of UV–vis features that match those obtained for **1** ^**+**^ (Figure 7e). By contrast, the oxidation of **2** in the presence of excess BH_3_·THF showed no evidence of **1** ^**+**^. The two absorbances at 330 and 410 nm for **2** decreased in intensity and blue-shifted to 315 and 360 nm respectively, and a new broad absorbance centered at 525 nm was observed (Figure 7f). We tentatively assign these features to **2** ^**+**^, but we note that the absorptions are more attenuated than expected when compared to transitions calculated using TDDFT (Figure S10).

### Chemical Oxidation Studies

To further investigate the chemical and electronic structures of the electrochemically generated species observed in the CV traces, **3** was chemically oxidized using AgBF_4_. Attempts to oxidize **1** in thf with different silver salts (AgX, where X = BF_4_^–^, PF_6_^–^, BArF_24_^–^, OTf^–^, and NTf_2_^–^) with the open metal coordination site resulted in complicated mixtures and evidence of ligand decomposition via S–Me cleavage. In contrast, samples of **3** in MeCN are readily oxidized with one or two equivalents of AgBF_4_ (Scheme 1). The singly oxidized complex was synthesized by mixing a one-to-one equivalent of **3** and AgBF_4_ in MeCN for 30 min. Filtration of the mixture to remove Ag(0) and removing the volatiles resulted in a dark solid that could be extracted into benzene to yield a dark green solution. Dark green crystals of **3­(BF**_**4**_ **)** were isolated in good yields (70%) by layering concentrated benzene solutions with Et_2_O. The doubly oxidized complex **3­(BF**_**4**_ **)**_**2**_ was prepared in good crystalline yield (73%) using a similar procedure by treating **3** with two equivalents of AgBF_4_ in MeCN. Removal of Ag(0) and layering a concentrated MeCN solution with Et_2_O resulted in dark red crystals of **3­(BF**_**4**_ **)**_**2**_ suitable for single-crystal XRD studies.

The structures of **3** and **3­(BF**_**4**_ **)** were reported previously,^23,24^ but the details of **3­(BF**_**4**_ **)** are described here for the first time for comparison to **3** and newly reported **3­(BF**_**4**_ **)**_**2**_. **3­(BF**_**4**_ **)** and **3­(BF**_**4**_ **)**_**2**_ adopt mononuclear structures with outer-sphere BF_4_^–^ anions. Overlaying the structures in Figure 8 shows that all the complexes have a distorted octahedral geometry about the Ru center. The Ru–N≡C angle becomes more linear for **3**, **3­(BF**_**4**_ **)**, and **3­(BF**_**4**_ **)**_**2**_, increasing in the order 169.6(4)°, 170.7(2)°, and 176.3(2)°, respectively. The N≡C bond distance in MeCN remains the same within error at 1.136(6), 1.131(6), and 1.137(5) Å, but the NC-Me distances shorten across the series from 1.472(8) Å in **3** to 1.457(4) Å and 1.447(5) Å for **3­(BF**_**4**_ **)** and **3­(BF**_**4**_ **)**_**2**_, respectively.

**Figure 8:**
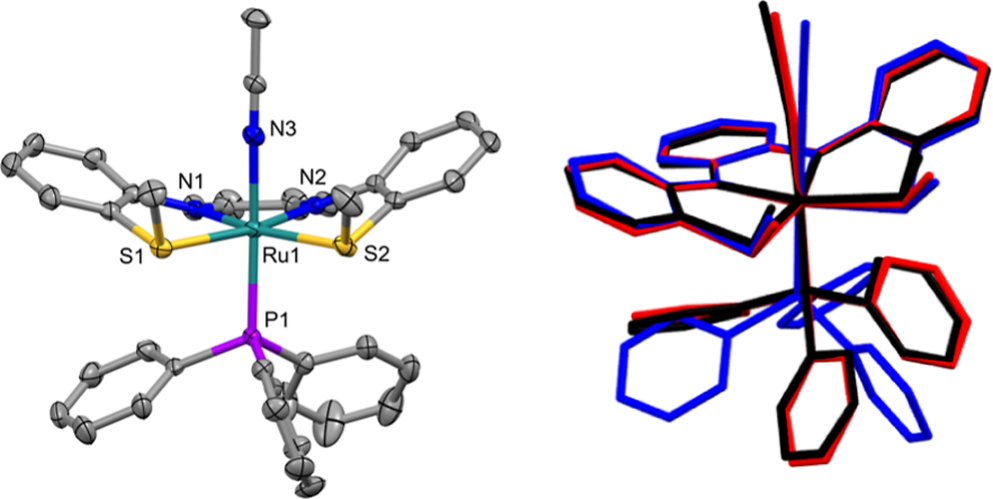
Left–molecular structure of **3­(BF**_**4**_ **)**_**2**_ with ellipsoids drawn at 50% probability level. Hydrogen atoms and the BF_4_^–^ anions were omitted from the figure. Right–structural overlay of **3** (black), **3­(BF**_**4**_ **)** (red) and **3­(BF**_**4**_ **)**_**2**_ (blue).

Examination of the **L1** bond distances reveals the changes expected for stepwise oxidation of the *o*-phenylenediamine backbone.^27^ Upon oxidation, the average N–C bond distances associated with the *o*-phenylenediamine subunit decreases from 1.391(7) Å in **3** to 1.376(3) and 1.345(3) Å in **3­(BF**_**4**_ **)** and **3­(BF**_**4**_ **)**_**2**_ corresponding to a stepwise increase in N–C double bond character (Table 5).^50,51^ The NC–CN bond correspondingly lengthens with oxidation from 1.442(6) Å in **3** to 1.460(4) Å in **3­(BF**_**4**_ **)**_**2**_, suggesting the formation of the *o*-diiminoquinone. Coincidingly, the average N–C bond distances of the flanking aryl groups reveal a stepwise increase with oxidation, indicating loss of N–C double bond character. These distances increase from 1.378(6) Å in **3** to 1.396(3) and 1.417(4) Å in **3­(BF**_**4**_ **)** and **3­(BF**_**4**_ **)**_**2**_.

**Table 5:**
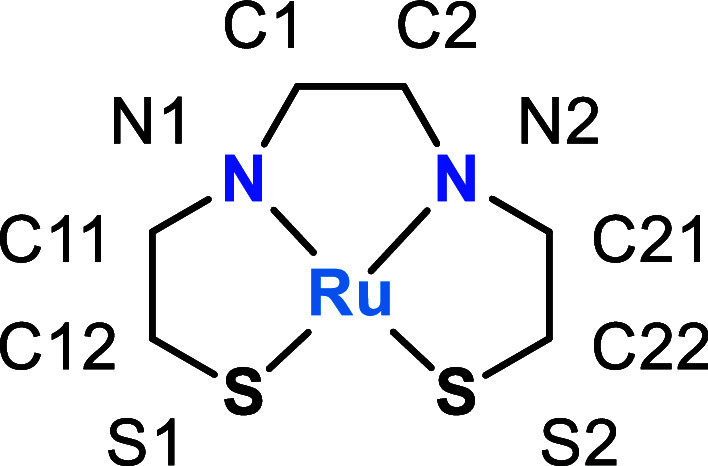
Select Bond Distances (Å) From Single-Crystal XRD Data

	1^a^	2^a^	3^a^	3(BF_4_)^a^	3(BF_4_)_2_
Ru–N1	2.006(6)	2.039(4)	2.034(3)	2.022(2)	1.997(2)
Ru–N2	2.017(5)	2.097(4)	2.046(4)	2.015(2)	1.993(2)
Ru–S1	2.330(2)	2.327(1)	2.330(1)	2.3502(7)	2.3741(8)
Ru–S2	2.338(2)	2.316(1)	2.323(1)	2.3416(7)	2.3766(8)
Ru–P	2.214(2)	2.317(1)	2.272(1)	2.3121(7)	2.3538(7)
Ru-X^b^		1.77(4)	2.136(3)	2.119(2)	2.084(2)
C1–C2	1.45(1)	1.418(7)	1.442(6)	1.438(4)	1.460(4)
N1–C1	1.39(1)	1.385(5)	1.390(6)	1.376(3)	1.348(3)
N2–C2	1.39(1)	1.465(6)	1.391(7)	1.376(3)	1.341(3)
N1–C11	1.39(1)	1.374(6)	1.378(5)	1.393(3)	1.415(4)
N2–C21	1.40(1)	1.452(6)	1.378(6)	1.398(3)	1.418(3)

a Reported previously in refs 23 and 24 .

b X = MeCN or H.

The Ru-L bond distances are also significantly affected by the changes in oxidation state, but they change in different directions depending on the hard/soft character of the donor atom. The softer Ru–S and Ru–P bonds lengthen upon oxidation whereas the three Ru–N distances associated with **L1** and MeCN become shorter. The average Ru–S distance increases from 2.327(1) Å to 2.3462(8) Å to 2.3754(8) Å for **3**, **3­(BF**_**4**_ **)** and **3­(BF**_**4**_ **)**_**2**_, respectively, mirroring the stepwise change observed in the Ru–P bond distances (2.272(1) to 2.3121(7) Å and finally to 2.354(1) Å). In contrast, the average Ru–N distances associated with **L1** decreases with oxidation from 2.040(4) Å to 2.019(2) Å to 1.995(2) Å. This follows the same trend in the Ru–MeCN distances, which decrease from 2.136(3) to 2.119(2) and finally to 2.084(3) for **3**, **3­(BF**_**4**_ **)** and **3­(BF**_**4**_ **)**_**2**_, respectively.

### Resonance Spectroscopies and Magnetic Susceptibility Measurements

^1^H, ^13^C, and ^31^P NMR spectra collected on **3­(BF**_**4**_ **)** revealed no resonances suggesting that the coordination cation is NMR silent due to its paramagnetism. Only ^11^B and ^19^F NMR signals pertaining to the outer sphere BF_4_ anion were observed at δ −0.42 and −150.33 ppm, respectively. In contrast, NMR analysis of **3­(BF**_**4**_ **)**_**2**_ revealed that this complex appears diamagnetic in solution, which initially suggested that the electronic structure of the ligand resembles a closed shell diiminoquinone. The room temperature ^1^H NMR spectrum in CD_2_Cl_2_ revealed single resonances at δ 2.05 for the methyl hydrogens of the coordinated MeCN and at δ 2.99 ppm the SMe groups indicating their equivalence on the NMR time scale. Three resonances assigned to the PPh_3_ ligand were observed in the aryl region at δ 6.95, 7.21, and 7.38 ppm. A very broad feature was observed in the baseline underneath the PPh_3_ resonances, which suggested some fluxionality in the triaryl N_2_S_2_ framework. Indeed, variable temperature ^1^H NMR spectra collected down to −80 °C resulted in sharpening of the broad aryl resonances and appearance of the correct number of resonances and integrations for the triaryl framework (Figure S4). The room temperature ^31^P NMR spectrum of **3­(BF**_**4**_ **)**_**2**_ in CD_2_Cl_2_ also yielded a sharp singlet at δ 36.7 ppm. For comparison, the ^31^P resonance for **3** in C_6_D_6_ in the presence of several drops of MeCN (to ensure bound MeCN) was δ 25.4 ppm.^23^ The ^11^B and ^19^F NMR spectra confirmed the presence of the outer sphere BF_4_ anions with resonances at δ −1.4 ppm for ^11^B and δ −152.8 ppm for ^19^F.

Despite the appearance of diamagnetism by NMR spectroscopy, subsequent magnetic susceptibility and EPR measurements on **3­(BF**_**4**_ **)**_**2**_ suggested the presence of unpaired spin, similar to that reported previously for doubly oxidized Ni complexes with **L1**.^27^ Anomalous diamagnetic NMR spectra have been observed previously in Ru complexes containing oxidized redox active ligands,^52,53^ as well as other complexes, despite the presence of unpaired spin.^54^ Notably, this has been observed in numerous complexes containing two semiquinone radicals. The radicals in these complexes were said to exhibit strong antiferromagnetic exchange mediated by filled d π orbitals that were strongly mixed with the semiquinone π orbitals.^55^

Magnetic susceptibilities of **3­(BF**_**4**_ **)** and **3­(BF**_**4**_ **)**_**2**_ were measured using the Evans method,^56^ and the results are compiled in Table 6 alongside the theoretical spin-only values for doublet (*S* = 1/2) and triplet (*S* = 1) electron configurations for comparison. The RT effective magnetic moment of singly oxidized **3­(BF**_**4**_ **)** was 2.2(1) μ_B_, which is higher than the spin-only value of 1.73 μ_B_ expected for a single unpaired electron (*S* = 1/2). The measurements on doubly oxidized **3­(BF**_**4**_ **)**_**2**_ also revealed an appreciable amount of unpaired spin with a μ_eff_ = 1.6(2) μ_B_. Variable temperature Evans method measurements collected on **3­(BF**_**4**_ **)** revealed ideal Curie–Weiss behavior where the μ_B_ spin value increased to a maximum 3.3 μ_B_ at −80 °C (Figure S5). Performing similar experiments for **3­(BF**_**4**_ **)**_**2**_ instead revealed temperature independent paramagnetism (TIP) where the μ_B_ spin value was effectively unchanged at the lowest temperature.

**Table 6:** Evans Method Magnetic Susceptibility Values for Singly- and Doubly-Oxidized 3 Reported in Units of Bohr Magnetons (μ_B_)­^a^

singly oxidized		doubly oxidized	
**3(BF**_**4**_ **)**		**3(BF**_**4**_ **)**_**2**_	
RT	2.2(1)	RT	1.6(2)
–80 °C	3.3	–80 °C	1.7
theoretical (S = 1/2)	1.73	theoretical (S = 1)	2.83

a Values shown at 20 °C (RT) are averages of triplicate measurements.Values shown at −80 °C are from variable T studies performed in CD_2_Cl_2_. Theoretical spin-only values are included for comparison,

EPR data were collected to determine the distribution of unpaired electron spins in both **3­(BF**_**4**_ **)** and **3­(BF**_**4**_ **)**_**2**_. Experiments reported previously on **3­(BF**_**4**_ **)** at 4 K confirmed the presence of a radical with significant electron spin delocalized across Ru and **L1** (Figure 9; top).^24^ The EPR spectrum was modeled as an *S* = 1/2 system with an isotropic signal at *g* = 2.0057 and *A* ^Ru^ = 74 MHz. The g value is close to that of a free electron, as expected for a ligand-centered radical, but the *A* ^Ru^ value suggests there is a nonnegligible ruthenium component (^101^Ru, *I* = 5/2; ^99^Ru, *I* = 3; natural abundance 16.98% and 12.8%, respectively). For comparison, typical *g* values for Ru­(III) can range from 2.033 to 2.205 ^57^ and would show the expected rhombohedral signal for a metal based radical as is the case with open-shell metal systems at 4 K.^58^ While no other hyperfine interactions could be detected due to line broadening, the relatively large hyperfine coupling of the Ru component at 2.63 mT is larger than what has been observed in similar systems.^59^

**Figure 9:**
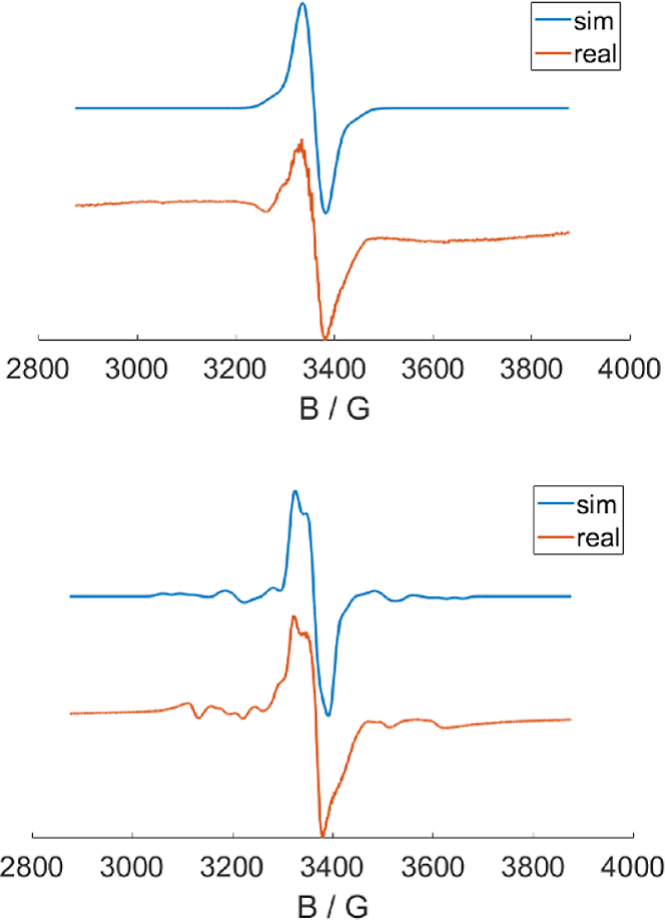
Frozen solution EPR spectra of **3­(BF**_**4**_ **)** (top) and **3­(BF**_**4**_ **)**_**2**_ (bottom) in CH_2_Cl_2_ at 4K in the X-band. The spectrum of **3­(BF**_**4**_ **)** was reported previously^24^ but is included here for comparison. Fitting parameters for **3­(BF**_**4**_ **)**: LW 5.4635, *g* = 2.0057, A^Ru^ = 74.2298 MHz. Fitting parameters for **3­(BF**_**4**_ **)**_**2**_: LW 2.5, *g*_1_ = 1.984, *g*_2_ = 2.0035, *g*_3_ = 2.0274, A_1_^Ru^ = 211.9 MHz, A_2_^Ru^ = 336.4, A_3_^Ru^ = 208.5 MHz.

EPR data collected for **3**(**BF**_**4**_)_**2**_ at 4 K revealed the presence of electron spin, indicating that the unpaired spin observed at higher temperature in the Evans measurements persists to some extent at this lower temperature (Figure 9; bottom). The EPR spectrum was modeled as an *S* = 1 system with a very slightly distorted rhombohedral signal with *g*_*1*_ = 1.984, *g*_*2*_ = 2.0035, and *g*_*3*_ = 2.0274. A nonnegligible amount of spin localization on Ru can be observed by the hyperfine Ru splitting with *A*_1_^Ru^ = 211.9 MHz, *A*_2_^Ru^ = 336.4, and *A*_3_^Ru^ = 208.5 MHz.

### Electronic Spectra of the Chemically Oxidized Complexes

Because of their limited solubility in MeCN and thf, the room temperature UV–vis spectra of **3­(BF**_**4**_ **)** and **3­(BF**_**4**_ **)**_**2**_ were recorded in CH_2_Cl_2_ (same solvent as EPR studies). The spectrum of **1** was also recollected in CH_2_Cl_2_ for comparison in the same solvent. The data are summarized in Table 1, and the overlaid spectra are shown in Figure S8. The spectrum of **1** in CH_2_Cl_2_ is nearly identical to the spectrum of **1** collected in thf, revealing intense absorptions at 264, 356, and 601 nm (37,878 cm^–1^, 28,090 cm^–1^ and 16,639 cm^–1^). Likewise, the spectra of the oxidized complexes are similar to those observed in the SEC studies of **3** conducted in MeCN. The higher energy UV absorptions observed in **1** are attenuated in **3­(BF**_**4**_ **)** and numerous new absorptions appear in the visible range between 500 and 750 nm. The absorptions at 510, 602, and 707 nm in **3­(BF**_**4**_ **)** occur at nearly identical energies to those observed in SEC studies of electrochemically generated **3** ^**+**^ in MeCN at 510, 605, 710 nm. Similar observations are made when comparing **3­(BF**_**4**_ **)**_**2**_ in CH_2_Cl_2_ and electrochemically generated **3** ^**2+**^ in MeCN. The spectrum for **3­(BF**_**4**_ **)**_**2**_ reveals a high intensity absorbance at 546 nm (ε = 14.4 × 10^3^ M^–1^ cm^–1^) and a slightly lower intensity feature at 428 nm (ε = 8.7 × 10^3^ M^–1^ cm^–1^). An LLCT band is again observed at the edge of the NIR window just beyond 900 nm.

XAS spectra were collected on **3­(BF**_**4**_ **)** and **3­(BF**_**4**_ **)**_**2**_ to further evaluate how oxidation effects the electronic structure of the unoccupied and single-occupied orbitals (Figure 10). As observed for S K-edge XAS data collected on **1**–**3**, the oxidized complexes revealed only subtle differences similar to that observed for complexes reported by Shearer and Grapperhaus.^47^ A slight increase in intensity and decrease in energy for the edge maxima as a function of oxidation suggests the orbitals involved in the redox processes contain little sulfur 3p character. The P K-edge XAS data, in contrast, yielded more pronounced differences. The prominent pre-edge feature at 2145.1 eV in the spectrum of **1** appears to blue shift under the rising edge at 2146.1 eV in **3­(BF**_**4**_ **)** based on the increase in intensity that is also observed for the second oxidation to **3**(**BF**_**4**_)_**2**_. However, a new low intensity pre-edge feature is observed at 2144.4 eV for **3**(**BF**_**4**_) and **3**(**BF**_**4**_)_**2**_.

**Figure 10:**
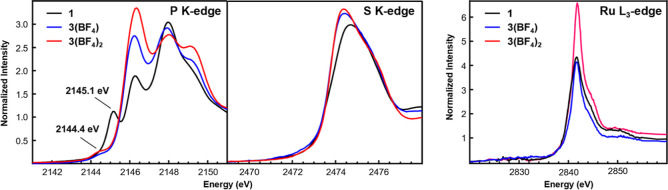
Comparison of normalized P K-edge, S K-edge, and Ru L_3_-edge XAS data for **1**, **3­(BF**_**4**_ **)**, and **3­(BF**_**4**_ **)**_**2**_.

The Ru L_3_-edge XAS data collected on **1**, **3­(BF**_**4**_ **)**, and **3­(BF**_**4**_ **)**_**2**_ proved to be the most informative with respect to changes in electronic structure as a function of oxidation state (Figure 10). Ru L_3_-edge transitions are primarily orbitally allowed Ru 2p→nd, and the spectra of formally Ru­(II) and Ru­(III) complexes yield diagnostic differences due to changes in oxidation state.^45,60,61^ For example, Ru­(III) complexes like [Ru­(NH_3_)_5_Cl]^2+^ and [Ru­(NH_3_)_6_]^3+^ yield two prominent features, a low energy peak at 2837.3–2837.4 eV and a higher energy peak 2840.9–2441.1 eV, whereas Ru­(II) complexes like [Ru­(bipy)_2_Cl_2_] and [Ru­(bipy)_3_]^2+^ only show the higher energy peak at 2840.3 and 2840.5 eV, respectively.^61^

The Ru L_3_-edge spectrum of **1** revealed a single prominent feature at 2841.6 eV, consistent with the formal oxidation state of Ru­(II). The energy of this feature is similar to the first absorption observed for [Ru^II^(CN)_6_]^4–^ at 2841.5 eV.^61^ Interestingly, no new peaks are observed at lower energy for **3­(BF**_**4**_ **)** and **3­(BF**_**4**_ **)**_**2**_, and the energy of the peak observed for **1** remains relatively unperturbed, indicating that there is no significant change in the formal Ru­(II) oxidation state as the complexes are oxidized. This suggests that the oxidations are primarily ligand centered, consistent with the stepwise changes observed in the **L1** N–C and NC–CN bond distances by XRD.

In contrast to the nearly identical Ru L_3_-edge peak positions, significant changes are observed in the peak intensities. The relative intensity of the edge feature for **3**(**BF**_**4**_) decreases slightly with respect to **1**, but both are significantly less intense compared to the edge peak observed for **3**(**BF**_**4**_)_2_. These intensity differences can be accounted for by changes in Ru 4d character in the associated MOs, as described in the following section.

### Electronic Structure Calculations for the Oxidized Complexes

Ground-state DFT calculations were performed on the singly and doubly oxidized complexes at the same level of theory described above for the neutral complexes. Calculations on the doubly oxidized complexes **1** ^**2+**^ and **3** ^**2+**^ were performed for both the closed-shell singlet and triplet configurations (**2** ^**2+**^ was not considered since it is not accessible, even with SEC measurements). The singlet configuration was lowest in energy for the doubly oxidized complexes with the singlet–triplet energy gap being 4.3 and 8.9 kcal/mol for **1** and **3**, respectively. DFT is a single-reference method; therefore, best-practices are to assess how well such a method performs for open-shell singlet diradicals by comparison with multireference methods.^62^ Note that open-shell singlet states were sought after at the DFT level, but were not found via conventional strategies. Therefore, single point energies were computed on **3** ^**2+**^ using complete active space self-consistent field (CASSCF) calculations with second-order perturbation theory (CASPT2). While the values support a ground state-singlet, the singlet–triplet energy gap is larger at 21.4 kcal/mol.

Two conclusions arose from these computations. First, the CASSCF singlet state is multiconfigurational with a percent radical character of 21%. Second, CASPT2 calculations were performed on both the DFT singlet and triplet optimized geometries. The singlet state is 2.8 kcal/mol lower on the triplet geometry compared to the singlet geometry supporting that the structural changes associated with additional radical character stabilize the singlet state. We attribute the stabilization arising from the “open-shell” character of the singlet state to the larger singlet–triplet splitting. We have focused the following comparisons on neutral and oxidized forms of **3** given that these complexes can be isolated experimentally, but the DFT calculations are qualitatively similar for oxidized forms of **1**.

The MO energies of **3**, **3** ^**+**^, and **3** ^**2+**^ are plotted in Figure 11 for comparison. The MOs decrease in energy as expected as **3** is oxidized to **3** ^**+**^ and then **3** ^**2+**^. The first oxidation removes an electron from the LUMO of **3** (2π). The second oxidation can occur by removing the second electron from the 2π to give the close-shell singlet configuration or by removing an electron from the 1π to give the triplet configuration. As shown in Figure 4, both the 2π and 1π MOs are Ru-L π* orbitals that are primarily ligand in parentage. They have relatively low Ru 4d character in neutral **3** (2π = 10% and 1π = 17%; Table 3), and the 4d percentages are similar in the oxidized complexes **3** ^**+**^ and **3** ^**2+**^ (Table 7). The lack of new MOs available with appreciable 4d character, as would be expected if there was metal-based oxidation of Ru­(II) to Ru­(III), accounts for why no new orbitally allowed 2p → 4d pre-edge features are observed in the Ru L_3_-edge XAS spectrum at lower energy. This indicates that both oxidations are centered primarily on the ligand. In a similar vein, the ligand-based 2π and 1π MOs have little in the way of P and S 3p character (Table 7). As shown below, the dearth of P and S 3p character in these MOs accounts for the lack of new features in the ligand K-edge XAS data as the 2π and 1π becomes available for transitions upon oxidation.

**Figure 11:**
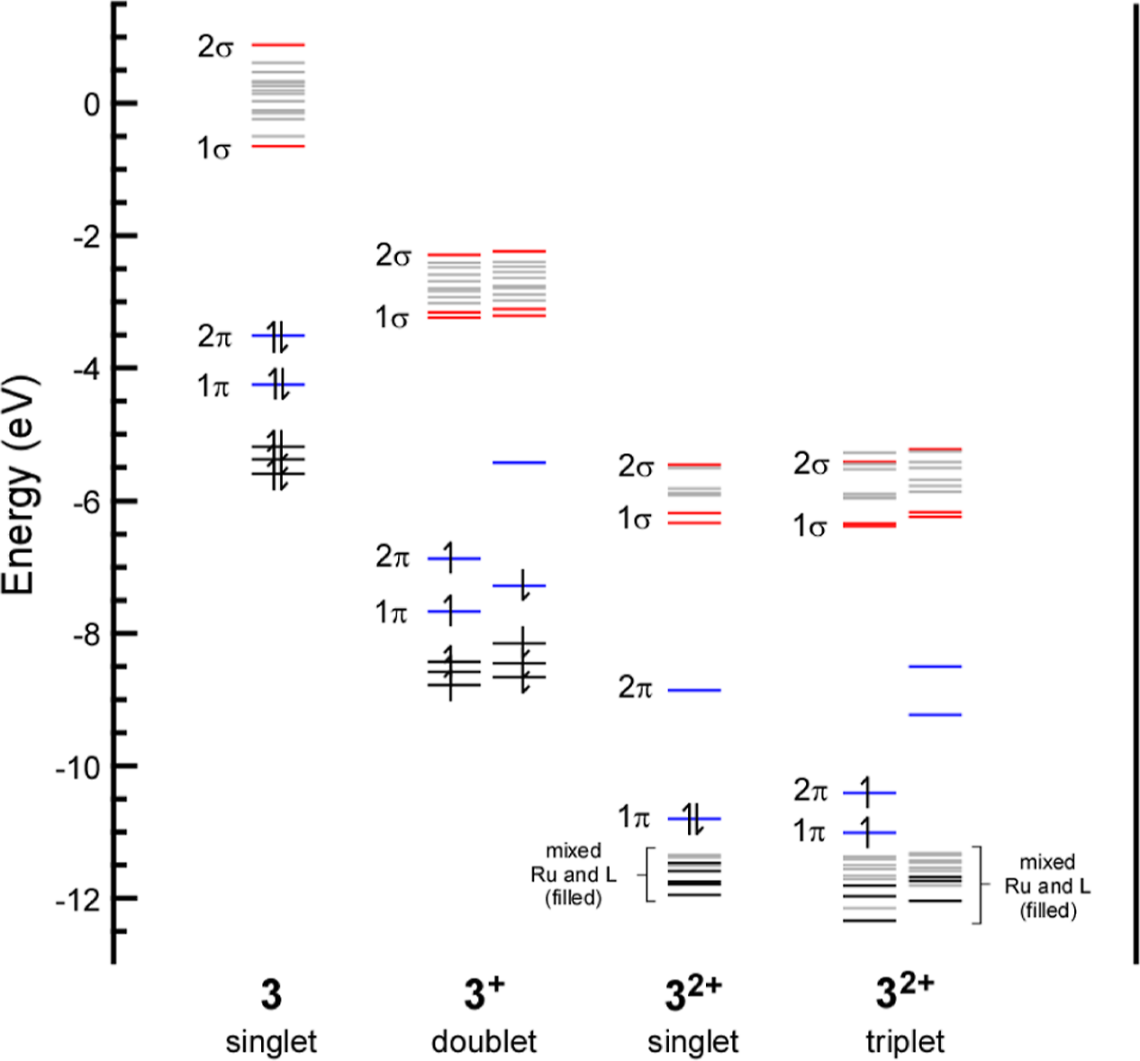
Truncated MO diagram for **3**, **3** ^**+**^, and **3** ^**2+**^ (singlet and triplet) from DFT calculations. Energies of the α and β orbitals are shown for the doublet and triplet configurations. The labels and MO color coding (blue = Ru-L π* and red = Ru-L σ*) matches that used for the qualitative MO diagram shown in Figure 4. Unoccupied orbitals that are derived of primarily ligand-based character are shown in gray.

**Table 7:** Calculated Compositions and Energies for Select Frontier MOs for 3^+^ and 3^2+^ ^a^

Complex	**MO**	**E (eV)**	**Ru (4d)**	**N (2p)** ^b^	**P (3p)**	**S (3p)**	assignment
**3** ^**+**^	191	–2.29	24%	6%		13%	d_*xy*_ (2σ)
(doublet)	182	–3.16	15%	2%	2%	8%	d_*xy*_
LUMO	181	–3.24	24%	2%	8%	4%	d_*z* ^2^_ (1σ)
SOMO	180	–6.87	12%	29%			d_*xz*_ (2π)
	179	–7.67	24%	20%		5%	d_*yz*_ (1π)
	178	–8.43	57%	2%		17%	d_*x*__^2^__‑*y*__^2^_
	177	–8.58	74%			2%	d_*xz*_
	176	–8.78	37%	2%	1%	1%	d_*yz*_
	175	–8.80	12%	1%	3%	2%	d_*yz*_
**3** ^**2**+^	187	–5.46	19%	5%	2%	10%	d_*xy*_ (2σ)
(singlet)	182	–6.18	36%	2%	11%	5%	d_*z* ^2^_ (1σ)
	181	–6.33	23%	4%		7%	d_*xy*_
LUMO	180	–8.86	14%	28%	1%		d_*xz*_ (2π)
HOMO	179	–10.80	21%	19%		5%	d_*yz*_ (1π)
	176	–11.47	13%	1%		5%	d_*xz*_
	174	–11.59	23%	1%		9%	d_*xz*_
	173	11.67	18%	3%		2%	d_*x*__^2^_-_*y*__^2^_
	172	–11.75	30%				d_*xz*_
	171	–11.79	44%	2%		4%	d_*x*__^2^__‑*y*__^2^_
	170	–11.95	44%	1%		1%	d_*yz*_
**3** ^**2+**^	188	–5.42	18%	5%	1%	8%	d_*xy*_ (2σ)
(triplet)	182	–6.34	34%	3%	8%	6%	d_*z* ^2^_ (1σ)
	181	–6.38	31%	4%	5%	8%	d_*xy*_
SOMO	180	–10.41	15%	26%			d_*xz*_ (2π)
SOMO	179	–11.01	17%	18%		7%	d_*yz*_ (1π)
	172	–11.81	25%	1%		18%	d_*xz*_
	171	–11.97	80%			3%	d_*x*__^2^__‑*y*__^2^_
	169	–12.34	40%			2%	d_*yz*_

a MOs shown are those with ≥10% Ru 4d character. Assignments are provided based on the identity of the Ru 4d orbital mixing in the MO and the labels provided in Figure 3. Energies Provided for the Doublet and Triplet configurations are for α Orbitals. Energies for both α and β orbitals are plotted in Figure 11.

b N *p*-character calculated for **L1** only. Does not include MeCN. MOs with orbital composition less than 0.5% are shown as blanks.

The energy changes observed in the MO diagram in Figure 11 has significant consequences when considering Ru 4d character mixing in the Ru–P and Ru–S σ* MOs. The Ru 4d character ultimately governs the intensity of the Ru L_3_-edge XAS features for **1**, **3­(BF**_**4**_ **)**, and **3­(BF**_**4**_ **)**_**2**_ given the orbital selection rules responsible for allowed Ru L_3_-edge transitions (Ru 2p → nd). The calculations indicate that **1** has a combined 64% Ru 4d character in the 1σ and 2σ MOs. Binding of acetonitrile and oxidation to **3** ^**+**^ yields an additional MO that has significant Ru 4d_*xy*_ character (15%) just above the 1σ (this MO is highlighted in red like the 1σ and 2σ orbitals in Figure 11). Taking the sum of these three MOs yields a combined Ru 4d character of 63%, which is similar to **1**, and consistent with their similar Ru L_3_-edge intensities. However, further oxidation to **3** ^**2+**^ yields an increase in the Ru 4d character in these three MOs to 77% (singlet) or 82% (triplet), which appears to account for the increased edge intensity observed in the Ru L_3_-edge XAS spectrum of **3­(BF**_**4**_ **)**_**2**_ relative to **1** and **3­(BF**_**4**_ **)** (Figure 10).

### TDDFT Calculations for Oxidized Complexes

TDDFT calculations corroborate the observed experimental spectra obtained for the oxidized complexes, and they lend further support to the electronic structure interpretation described above based on DFT. Starting with the UV–vis spectra, removing electrons from the 2π and 1π MOs upon oxidation yields additional low energy features assigned to MLCT transitions into these newly available orbitals. The energies and relative intensities of the calculated transitions for **1** ^**+**^ and **3** ^**+**^ are in good agreement with the absorptions observed in the SEC measurements (Figures 12 and S11). For the doubly oxidized complexes **1** ^**2+**^ and **3** ^**2+**^ both singlet and triplet configurations were considered in the TDDFT calculations. Both configurations yield transitions that are relatively consistent with the experimental spectra, but the triplet configuration for **1** ^**2+**^ appears to capture more of the weak absorptions at lower energy (10,000–15,000 cm^–1^). Higher energy transitions are also calculated for **2** ^**+**^ (Figure S10), but they do not appear to match as well as those for **1** ^**+**^ and **3** ^**+**^. The experimental absorptions at higher energy are more attenuated than calculated for **2** ^**+**^, which may be attributed to experimental concentrations of **2** ^**+**^ being lower than expected due to its equilibrium with BH_3_ in solution.

**Figure 12:**
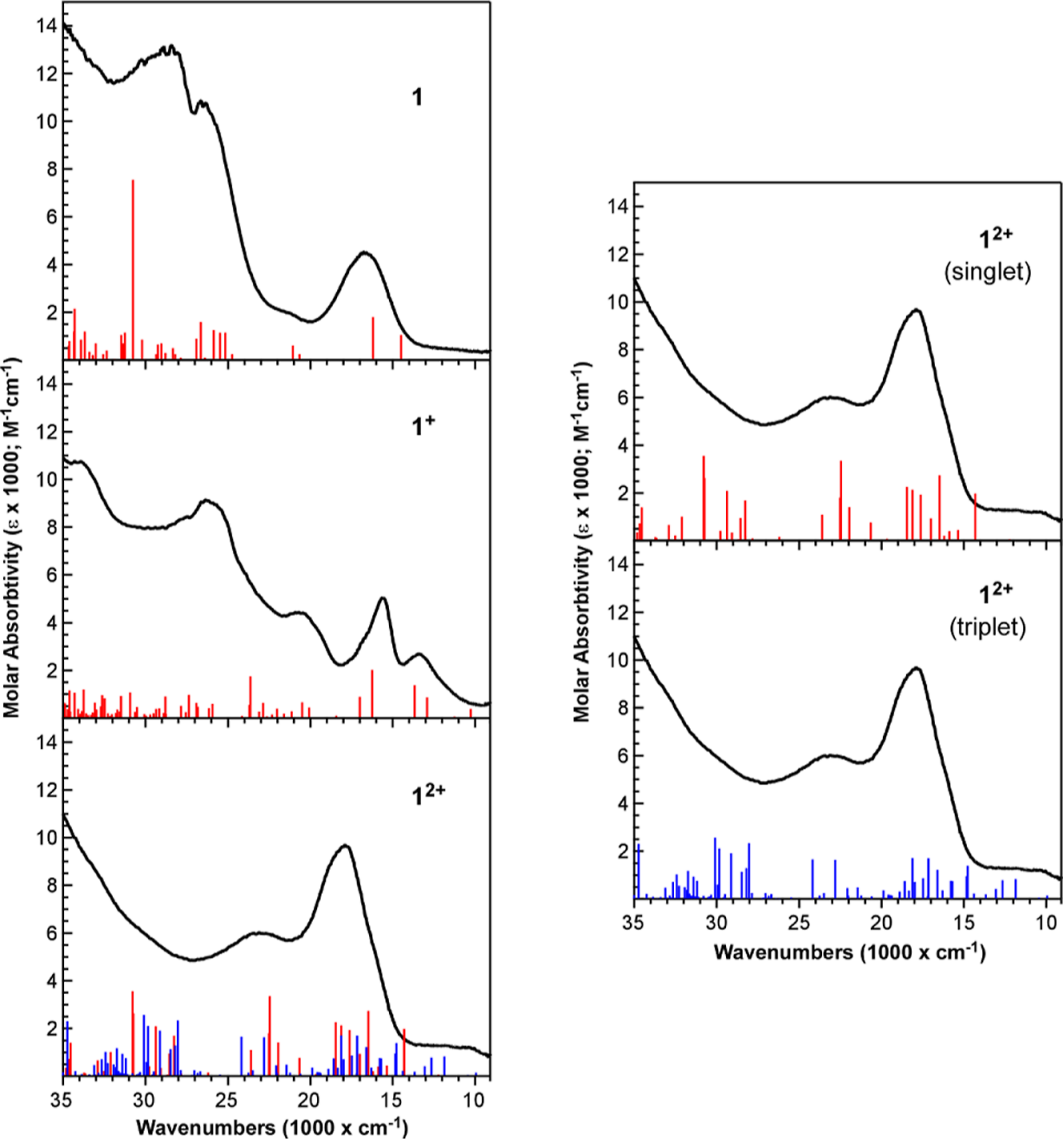
Comparison of the UV–vis SEC spectra of **1**, **1** ^+^, and **1** ^2+^ to calculated transitions from TDDFT. The plot of doubly oxidized **1** ^2+^ on the left shows TDDFT transitions for both the calculated singlet (red) and triplet (blue) configurations. The plots on the right compare the calculated singlet and triplet transitions separately for comparison. Calculated oscillator strengths for the transitions have been multiplied by a factor of 5.0 × 10^4^ to bring them on scale with the experimental results.

TDDFT calculations were also performed at the P and S K-edge so the simulated and experimental ligand K-edge XAS spectra could be compared for **3** ^**+**^ and **3** ^**2+**^. As shown for the neutral complexes, the simulated spectra again reproduce the most salient experimental features (Figures 13 and S13). The newly accessible 2π and 1π MOs in **3­(BF**_**4**_ **)** and **3­(BF**_**4**_ **)**_**2**_ have minimal S and P 3p character, which accounts for the lack of prominent (i.e., orbitally allowed 1s → 3p) pre-edge features in the P and S K-edge XAS spectra as **3** undergoes stepwise oxidation. Indeed, the TDDFT calculations predict a weak transition assigned as P 1s → 2π in the pre-edge baseline in the simulated P K-edge XAS spectrum of **3** ^**+**^ at 2144.0 eV that becomes more perceptible in the spectrum of singlet **3** ^**2+**^ at 2143.4 eV (marked with arrows in Figure 13). This likely corresponds to the weak pre-edge absorptions observed in the P K-edge XAS spectra of **3­(BF**_**4**_ **)** and **3­(BF**_**4**_ **)**_**2**_ at 2144.4 eV. Similar pre-edge transitions are harder to observe in the experimental and simulated S K-edge XAS spectra of **3­(BF**_**4**_ **)** and **3­(BF**_**4**_ **)**_**2**_ (Figure S13). As with the TDDFT calculations for the UV–vis spectrum of triplet **3** ^**2+**^, the calculated ligand K-edge XAS spectra show only relatively small differences when compared to those for singlet **3** ^**2+**^.

**Figure 13:**
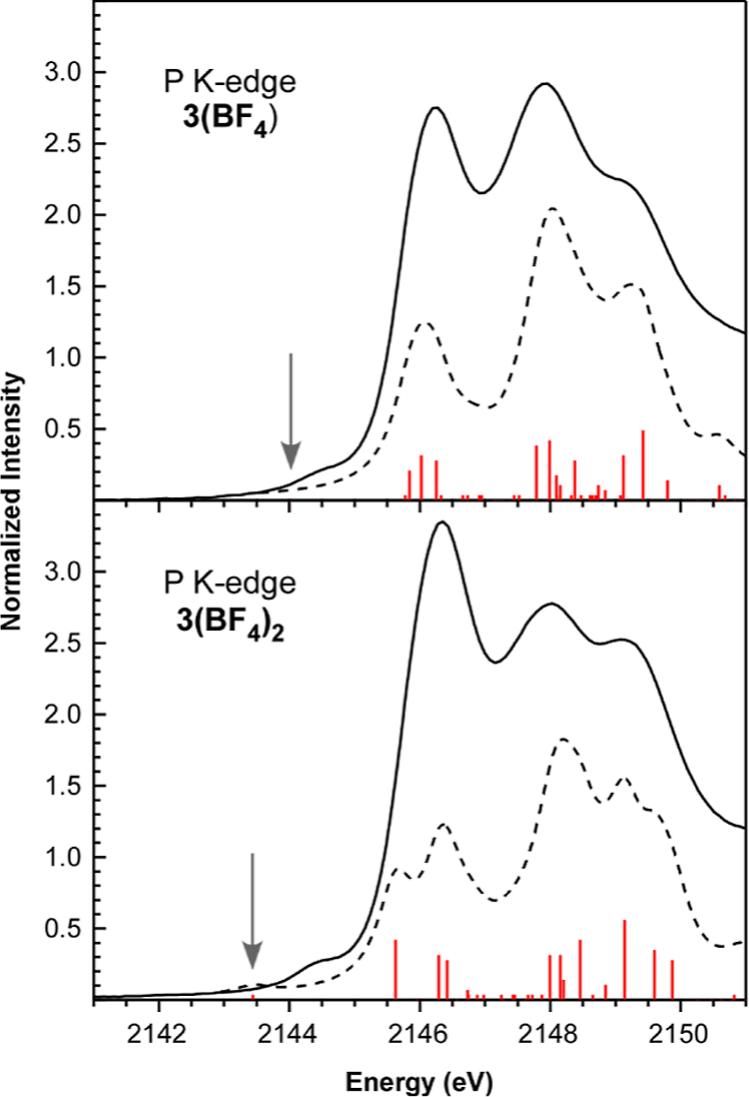
Comparison of experimental and simulated P K-edge XAS data for **3** ^**+**^ and **3** ^**2+**^ (closed-shell singlet). The experimental spectra (solid lines), simulated spectra (dashed lines), and calculated transitions (red bars) are shown. Calculated oscillator strengths were multiplied by a factor of 350 to bring them on scale with the experimental data. An energy shift of +49.7 eV (P K) was applied to the calculated spectra so that relative differences in calculated and experimental peak positions could be compared. The gray arrows are included to emphasize pre-edge transitions associated with the 2π MOs.

## Discussion

The spectroscopic, structural, and theoretical data appear to converge on a consistent picture of the electronic structure in neutral and oxidized species of **1**, **2**, and **3**. To facilitate the following discussion, Figure 14 is provided to show some of the more relevant formal metal and ligand oxidation states possible.

**Figure 14:**
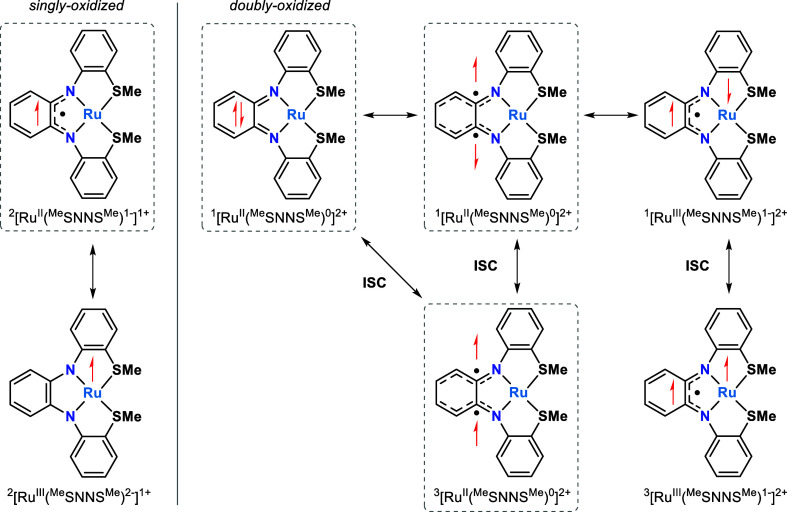
Formal oxidation states and select resonance forms possible for singly oxidized **3** ^**+**^ (left) and doubly oxidized **3** ^**2+**^ (right). Axial PPh_3_ and MeCN ligands are not shown. ISC = intersystem crossing. Resonance forms representing ligand-centered oxidations are emphasized with dashed boxes.

Starting with the analysis of the singly oxidized species **1** ^**+**^ and **3** ^**+**^, there are two possible resonance forms to be considered based on the locus of oxidation: metal-based oxidation can result in Ru^III^(**L1**)^2–^ while ligand-based oxidation results in Ru^II^(**L1** ^**•**^)^1–^. Analysis of ligand bond distances from single-crystal XRD collected on **3­(BF**_**4**_ **)** and the relative position of the *g* value from EPR spectroscopy at *g* = 2.0057 suggest that the major resonance form is likely a ligand based radical with a Ru^II^ center. However, we note there is a significant Ru spin component indicative of an efficient spin transfer (spin polarization) from the *o*-diiminosemiquinone radical of **L1** to the Ru center, as is well established with chelating noninnocent ligands containing strong Ru^II^–N bonds.^14,63^ The efficient spin transfer is further supported by the paramagnetic character displayed in the variable temperature Evans method measurement of **3­(BF**_**4**_ **)**. DFT calculations lend further support for this assessment by showing that the SOMO is ligand-derived Ru–N* (i.e., mostly ligand in character) due to the inverted bonding between Ru 4d and the filled **L1** π orbitals localized on the *o*-benzosemiquinonediiminate subunit (Figure 4). The XAS and UV–vis (SEC) data are in good agreement with simulated transitions and spectra from TDDFT, which suggests that the DFT calculations are effectively modeling the electronic structure. We note that the coordination of MeCN in the axial site trans to PPh_3_ does not result in valence tautomerism, as observed previously with oxidized **L1** and Ni­(II).^27^

The doubly oxidized complexes **1** ^**2+**^ and **3** ^**2+**^ have the possibility of more formal resonance forms due to singlet and triplet configurations. The simplest configuration possible for the doubly oxidized complexes is Ru^II^ with a neutral, closed-shell **L1** with a *o*-benzoquinonediimine subunit. This configuration is consistent with the observed changes in **L1** bond distances in the structure of **3­(BF**_**4**_ **)**_**2**_ but does not reconcile the magnetic susceptibility and EPR data suggesting the presence of unquenched spin. We therefore consider the resonance structures that have unpaired electrons. The Ru L_3_-XAS data suggests that the oxidation state remains Ru^II^ in **3­(BF**_**4**_ **)**_**2**_, thereby ruling out any resonance structures that invoke Ru^III^ (or Ru^IV^, which is not shown in Figure 14, but is also sometimes considered).^14^ This leaves the open-shell singlet and triplet configurations of [Ru^II^(**L1** ^**••**^)^0^(PPh_3_)]^2+^ and [Ru^II^(**L1** ^**••**^)^0^(PPh_3_)­(MeCN)]^2+^ as possible candidates. DFT calculations indicate that the triplet configuration is significantly higher in energy than the closed-shell singlet, and too high in energy to be thermally populated to any appreciable extent. CASSCF calculations corroborate these findings and further indicate that the ground state is multiconfigurational with 21% singlet diradical character.

The NMR data collected for **3­(BF**_**4**_ **)**_**2**_ also suggests a diamagnetic complex consistent with the calculations despite the magnetic susceptibility and EPR data indicating presence of unpaired spin. However, this dichotomy–diamagnetic-appearing NMR spectra obtained for paramagnetic complexes–has been reported previously in complexes with noninnocent ligands,^52,53^ perhaps most notably with the bis–semiquinone complex *cis*-Ru­(PPh_3_)_2_(1,2-O_2_C_6_H_4_)_2_.^53^ The anomalous diamagnetic NMR spectrum was attributed to a singlet–triplet equilibria and a singlet–triplet gap of 4.2–4.3 kcal/mol in toluene, which is similar to that calculated here for **1** ^**2+**^ (but lower than **3** ^**2+**^). This singlet–triplet energy difference was measured experimentally by monitoring the change in aryl ^1^H chemical shifts as a function of temperature using variable-temperature NMR spectroscopy.^53^ Brown and co-workers modified this approach slightly to measure higher energy singlet–triplet gaps in group 10 bis­(iminosemiquinone) complexes.^54^ Unfortunately, it is difficult to assess the singlet–triplet gap in **3­(BF**_**4**_ **)**_**2**_ for comparison to the calculated energies because of fluxional broadening of the **L1** aryl groups on the NMR time scale (Figure S4).

A final observation with relevance to the discussion here is the temperature independent paramagnetism (TIP) observed for **3­(BF**_**4**_ **)**_**2**_ down to −80 °C. TIP can be observed when there is singlet–triplet mixing due to spin–orbit coupling, and it has been invoked to account for anomalous ^1^H NMR shifts that appeared more diamagnetic than expected in Fe complexes containing ligand-centered radicals in redox noninnocent 2,6-diiminepyridine (PDI) ligands.^64^ Similar anomalous ^1^H NMR shifts were reported for Co complexes with PDI ligands,^65^ but the authors noted that it was difficult to discern if they were attributed to TIP (i.e., singlet–triplet mixing), thermal population of the triplet state, or some combination of both.^66^ Singlet–triplet mixing, as reflected by the experimentally observed TIP, appears more likely to account for the observations reported here for **3­(BF**_**4**_ **)**_**2**_ based on the following: (1) increased spin–orbit coupling expected for Ru (as compared to first–row transition metals), (2) the relatively large singlet–triplet gap calculated for **3** ^**2+**^ using DFT and CASPT2, and (3) the unquenched spin observed by EPR spectroscopy at 4 K.

## Conclusion

Our investigation of Ru complexes containing redox-active **L1** revealed the underlying electronic structure of the neutral and oxidized complexes and established the locus of oxidation. Though we were unable to isolate **1** in its oxidized form, UV–vis spectroelectrochemistry (SEC) yielded spectra that were in good agreement with DFT and TDDFT calculations for the putative species **1** ^**+**^ and **1** ^**2+**^. SEC analysis of **2** with BH_3_ bound in a metal–ligand cooperative (MLC) fashion revealed immediate BH_3_ loss upon oxidation to form **1** ^**+**^ and showed how this can be attenuated by running the experiments in the presence of excess BH_3_·thf. In contrast to **1** and **2**, we described how MeCN-bound **3** was isolable in its singly and doubly oxidized forms for structural characterization and ex-situ analysis. The experimental data collected for **3­(BF**_**4**_ **)** and **3­(BF**_**4**_ **)**_**2**_ again revealed relatively good agreement with the theoretical results, inspiring confidence in the information gleaned from (TD)­DFT calculations on the nonisolable species.

The experimental and theoretical results indicate that the first two oxidations of **1** and **3** are centered on the *o*-phenylenediamide backbone of the **L1** ligand. Ru L_3_-edge XAS studies on oxidized samples of **3**, for example, confirmed that Ru is not oxidized and remains in the +2-oxidation state during sequential oxidation. DFT calculations indicated that the highest occupied molecular orbitals involved in the oxidations are primarily of ligand parentage. TDDFT calculations showed good agreement with the UV–vis and ligand K-edge XAS spectra collected for the neutral and oxidized complexes, suggesting that the calculations were modeling the electronic structure effectively.

Perhaps the most unusual finding concerns the electronic structure of doubly oxidized **3­(BF**_**4**_ **)**_**2**_. EPR and magnetism studies of this complex revealed the presence of unpaired spin, which was somewhat unexpected given the diamagnetic appearance of the NMR data and structural metrics consistent with a fully oxidized ligand backbone. DFT calculations indicate a singlet ground state and a triplet excited state that is likely too high in energy to populate to any appreciable extent (8.9 kcal/mol). CASPT2 calculations confirmed the singlet ground state and further indicated that the singlet state is multiconfigurational with 21% radical character stemming from the open-shell singlet configuration. The lack of a thermally accessible triplet configuration suggests that the unpaired spin in **3­(BF**_**4**_ **)**_**2**_ can be attributed to singlet–triplet mixing facilitated by Ru spin–orbit coupling, which is consistent with its temperature-independent paramagnetism.

Collectively, these findings are emblematic of how complementary spectroscopic, structural, and theoretical methods are often required to assign redox formalisms and fully elaborate the electronic structure of complexes containing noninnocent ligands in different redox states.

## Experimental Section

### General Considerations

All reactions were performed under an atmosphere of N_2_ or Ar using glovebox or standard Schlenk techniques, unless stated otherwise. Tetrahydrofuran (thf), diethyl ether (Et_2_O), acetonitrile (MeCN), and CH_2_Cl_2_ were dried and degassed using a Pure Process Technologies Solvent Purification System and stored over 3 Å molecular sieves for several days before use. Deuterated solvents were deoxygenated by three freeze–pump–thaw cycles and stored over 3 Å molecular sieves. **1**, **2**, **3**, and **3­(BF**_**4**_ **)** were prepared as described previously.^23,24^ All other chemicals were purchased from commercial vendors and used as received.

^1^H, ^11^B, and ^31^P NMR data were recorded on a Bruker DRX-400 instrument operating at 400 MHz for ^1^H, 376 MHz for ^19^F, 128 MHz for ^11^B, and 162 MHz for ^31^P. Chemical shifts are reported in δ units relative to residual solvent peaks (^1^H), 85% H_3_PO_4_ (^31^P; δ 0.0 ppm), BF_3_·Et_2_O (^11^B; δ 0.0 ppm), or 0.05% C_6_H_5_CF_3_ in C_6_D_6_ (^19^F; δ −62.9 ppm). Microanalytical data (CHN) were collected using an EAI CE-440 Elemental Analyzer in the UI Department of Chemistry. IR spectra were acquired on a Thermo Scientific Nicolet iS5 using an attenuated reflection accessory. UV–vis absorption data collected on isolated complexes were obtained using an Agilent Cary 5000 spectrometer. EPR spectra in the X band were recorded with a Bruker EMX System. The solution was frozen in liquid nitrogen and the sample was placed in the resonator at 4 K. Measurements were carried out with a frequency of 9.46 GHz. Spectra were simulated using EasySpin^67^ running on Matlab.

### 3­(BF_4_)_2_

AgBF_4_ (0.028 g, 140 mmol) in MeCN (5 mL) was added dropwise to a green-yellow solution of **3** (0.050 g, 70 mmol) in MeCN (10 mL). An immediate color change to dark purple-red was observed and the solution was allowed to stir for 30 min. The mixture was filtered over a Celite pad to remove Ag^0^ and concentrated by removing some of the solvent under vacuum. Layering with Et_2_O yielded in dark red needles. Yield: 0.047 g (73%). Anal. Calcd For C_40_H_36_B_2_F_8_N_3_PRuS_2_: C, 51.74; H, 3.91; N, 4.53. Found: C, 51.88; H, 3.97; N, 4.51. ^1^H NMR (400 MHz, CD_2_Cl_2_, RT): δ 2.05 (br s, 3H, MeCN), 2.99 (br s, 6H, SMe), 6.95 (dd, *J* = 7.50 Hz, 7H, PPh_3_ Ar–H), 7.21 (t, *J* = 8.00 Hz, 8H, PPh_3_ Ar–H), 7.39 (t, *J* = 7.50 Hz, 4H, Ar–H). ^1^H NMR (400 MHz, CD_2_Cl_2_, −80 °C): δ 2.06 (br s, 3H, MeCN), 2.90 (br s, 6H, SMe), 6.86 (br s, 6H, PPh_3_ Ar–H), 7.15 (br s, 7H, PPh_3_ Ar–H), 7.33 (br t, *J* = 7.10 Hz, 4H, PPh_3_ Ar–H), 7.41 (br s, 6H, Ar–H), 7.50 (br s, 2H, Ar–H), 7.77 (br s, 2H, Ar–H). ^31^P NMR (162 MHz, CD_2_Cl_2_): δ 36.66. ^11^B NMR (128 MHz, CD_2_Cl_2_): δ −1.41. ^19^F NMR (376 MHz, CD_2_Cl_2_): δ −152.77, −152.82. IR (ATR, cm^–1^): 3080 (w), 2937 (w), 2876 (w), 2317 (w), 2287 (w), 1571 (m), 1518 (m), 1481 (m), 1458 (m), 1433 (s), 1365 (m), 1353 (m), 1304 (m), 1284 (m), 1163 (m), 1048 (vs), 1030 (vs), 997 (s), 951 (s), 871 (m), 844 (m), 755 (s), 742 (s), 692 (s), 657 (m), 638 (m).

### Single-Crystal XRD Studies

Single crystals of **3**(**BF**_**4**_)_**2**_ grown from MeCN/Et_2_O were mounted on a MiTeGen micromount with ParatoneN oil. The data were collected on a Bruker Nonius KappaCCD with an Apex II charge-coupled-device (CCD) detector. The sample was cooled to 150(2) K by an Oxford Cryostreams 700 low temperature device. The instrument was equipped with a graphite monochromatized MoKα X-ray source (λ = 0.71073 Å). A hemisphere of data was collected using phi and omega scans. Data collection, initial indexing, and cell refinement were conducted using the Bruker Apex II suite. The data were corrected for absorption using redundant reflections and the SADABS program.^68^ Structures were solved by direct methods and difference Fourier techniques performed in Olex2.^69^ Hydrogen atom positions were idealized and allowed to ride on the attached carbon atoms. A solvent mask^70^ was applied to the refinement to account for disordered MeCN solvent in the crystal lattice that could not be modeled satisfactorily. HKL reflections with error/esd values ±10 were omitted from the models. The final refinement included anisotropic temperature factors on all non-hydrogen atoms. Structure solution and refinement were performed in Olex2 using SHELXT and SHELXL, respectively.^71,72^ Graphics and publication materials were created in Mercury.^73^ Data collection and refinement details are listed in Table S1.

### Electrochemical Studies

Cyclic voltammetry (CV) experiments were performed under a N_2_ atmosphere in a Genesis glovebox or on the benchtop using an air-free cell. Data were collected using a CH Instruments CHI660D or a CH Instruments CHI1100B potentiostat. All glassware was cleaned by soaking in a bath of Nochromix acid overnight, rinsing with DI water, placing in a bath of nitric acid overnight, rinsing with DI water, and rinsing again with boiling milli-Q water, and drying the glassware at 150 °C in a dedicated glassware oven. The electrochemical solution was purged in the glovebox for 10 min with ultrahigh purity (UHP) Ar (99.999%) before each CV experiment was carried out. All analyte concentrations were 1 mM unless otherwise stated. The electrolyte used was 0.1 M (^*n*^Bu_4_N)­PF_6_ (Aldrich, ≥99.0%) in MeCN or thf unless otherwise stated. MeCN and thf were dried and deoxygenated using a Pure Process Technologies solvent system and stored over 3 Å molecular sieves before use.

### Spectroelectrochemistry

UV–vis SEC studies were carried out using the WaveNow potentiostat from Pine Research in bulk electrolysis mode in concert with the Avion UV–vis spectrophotometer from Pine Research. Solutions were prepared in the same manner as described for cyclic voltammetry measurements. In all cases, a three-electrode setup was used. The reference electrode was a Pt wire. A honeycomb ceramic electrode containing gold working and counter electrodes was used and placed in a 1.7 mm path length quartz cell designed for spectroelectrochemistry that was purchased from Pine Research. A blank spectrum was collected of the supporting electrolyte in the cell setup to account for the absorbance of the gold honeycomb electrode before data were collected. All solutions were degassed for approximately 10 min under UHP argon (99.999%) and collected in a nitrogen atmosphere glovebox. The various redox states were generated electrolytically in situ by applying potentials held for 100 s (to achieve bulk electrolysis) before obtaining UV–vis scans. UV–vis scans were obtained in 10 mV increments throughout the electrochemical window.

### Density Functional Theory Calculations

All calculations were performed with density functional theory as implemented by the Gaussian 09 computational chemistry suite.^74^ Starting geometries were informed by the crystal structures and all structures were fully optimized. Initial optimizations were performed at the B3LYP-d3 level.^75,76^ Ru was modeled with the effective core potential and uncontracted basis set of Hay and Wadt augmented with a set of f-orbital polarization functions collectively known as LANL08­(f).^77–79^ All other atoms were modeled with Pople’s split-valence double-ζ 6-31G­(d,p) plus polarization basis set.^80^

Wave function stability was verified to ensure that the electronic states being studied were correctly modeled.^81,82^ The existence of the correct number of imaginary frequencies was determined through evaluation of the analytic Hessian, which along with the potential energy and gradient information was used to generate the zero-point energy as well as enthalpy and free energy corrections to the internal energy.

Time-dependent density functional theory (TDDFT) calculations were combined with information on the charges, spin densities and relative orbital contributions from Mulliken population analysis^83^ of the individual molecular orbitals from the ground state structures in order to simulate and interpret the XAS. These methods have been employed previously to produce good agreement with experimental XAS data.^33–44^

### Computational Details for the CASSCF/CASPT2 Calculations

DFT optimized geometries were subjected to the multiconfigurational complete active space (CASSCF)^84^ single-point calculations followed by second-order perturbation theory (CASPT2).^85,86^ Two active spaces were used: (2e,2o) and (6e,6o). The (2e,2o) incorporated the π and π* orbitals of the ligand. A larger (6e,6o) also incorporated two pairs of M-L σ and σ* orbitals. Values calculated using the larger (6e,6o) active space is discussed in the main text, but data from the minimal (2e,2o) space calculations are included in the ESI to emphasize that correlating two electrons sufficiently recovers the correct multiconfigurational singlet state. In the CASPT2 calculations, the standard definition of the zero-order Hamiltonian (IPEA = 0.25 a.u) is used.^87^ To exclude the possible intruder states, an imaginary shift of 0.2 a.u was applied. The scalar relativistic effects were included at the CASSCF level using the second order Douglas-Kroll-Hess Hamiltonian^88^ and relativistic all electron ANO-RCC basis sets.^89^ The following contractions were used: [7s6p4d2f1g] for Ru, [4s3p2d1f] for N, [5s3p2d1f] for S, [4s3p1d] for P, [3s2p1d] for C, and [1s] for H atoms. Cholesky decomposition^90^ in conjunction with local-exchange screening was used to reduce the computational cost. These CASSCF and CASPT2 calculations are carried out using the OpenMolcas software suite.^91^ Orbitals shown in the ESI (Figures S14–S21) were plotted using Luscus with an isosurface value of 0.04.

### XAS Studies

XAS data were collected at the Stanford Synchrotron Radiation Lightsource (SSRL) under dedicated operating conditions of 3.0 GeV and 500 mA. XAS data were collected at Beamline 4–3 (S K-edge and Ru L_3_-edge) and 14–3 (P K-edge) with previously described beamline equipment, optics, and experimental conditions. Samples were prepared as described previously in an N_2_ or Ar glovebox and measured under a flow of ultrahigh purity He (99.999%) using a sample flow cell designed for XAS analysis of air-sensitive samples in the tender X-ray region.^42^ Sample fluorescence was measured using a Vortex 4-element Si detector or passivate implanted planar Si (PIPS) detector, and incident radiation (*I*_0_) was measured using a He ionization chamber. When used, the Vortex detector was placed at sufficient distance from the sample chamber window to ensure dead times were less than 3%. The first pre-edge features in the S K-edge XAS spectrum of Na_2_S_2_O_3_ (2472.02 eV)^29^ and the P K-edge spectrum of PPh_4_Br (2146.96 eV)^40^ were used to calibrate incoming X-ray energy. The Ru L_3_-edge data were calibrated using the first pre-edge feature in the Cl K-edge XAS spectrum of Cs_2_CuCl_4_ (2820.20 eV).^29^ Calibration scans were collected before and after triplicate runs of each sample. Calibrations, background subtractions, and normalizations were performed using the Athena program in the IFEFFIT XAS software suite.^92^ Normalizations were performed as previously described^27,40,42–44^ using set points of 2490 eV (S K-edge), 2165 eV (P K-edge), and 2860 (Ru L_3_-edge).

## Supplementary Material





## Notes

Tabulated crystallographic data and variable-temperature ^1^H NMR spectra for **3­(BF_4_)**_**2**_, experimental and calculated bond distances and angles from DFT. Comparisons of experimental and simulated spectra and transitions from TDDFT calculations. Singlet and triplet energies, CASSCF active natural orbitals, and effective bond orders from CASPT2 calculations (PDF)
Coordinates of calculated structures (XYZ)
