# Supplementary material for: Spectroscopic and Theoretical Studies of Ruthenium Complexes with a Noninnocent N2S2 Ligand in Different Redox States
Source: Inorg Chem. 2025 Jul 26;64(31):15961–79. doi: 10.1021/acs.inorgchem.5c02059 (PMC12344773; doi:10.1021/acs.inorgchem.5c02059)
Supplement: Supplementary file 1 [file ic5c02059_si_001.pdf]

SUPPORTING INFORMATION

Spectroscopic and Theoretical Studies of Ruthenium Complexes with a Noninnocent N_2S_2 Ligand in Different Redox States

Javier A. Luna,[1,†] Kyle D. Spielvogel,[1,†] Nathan R. Loutsch,[1] Sydney M. Loria,[2] Leah P. Weisburn,[2] Mark R. Ringenberg,[3] Bess Vlaisavljevich,[1] Jason M. Keith,[2,*] Scott K. Shaw,[1,*] Scott R. Daly[1,*]

[†]*Co-first authors*

[1]*Department of Chemistry, The University of Iowa, E331 Chemistry Building, Iowa City, Iowa 52242-1294, United States*

[2]*Department of Chemistry, Colgate University, 13 Oak Drive, Hamilton, New York 13346, United States*

[3]*Universität Stuttgart, Institut für Anorganische Chemie, Pfaffenwaldring 55, 70569 Stuttgart, Germany*

Corresponding emails: scott-daly@uiowa.edu, scott-k-shaw@uiowa.edu, jkeith@colgate.edu

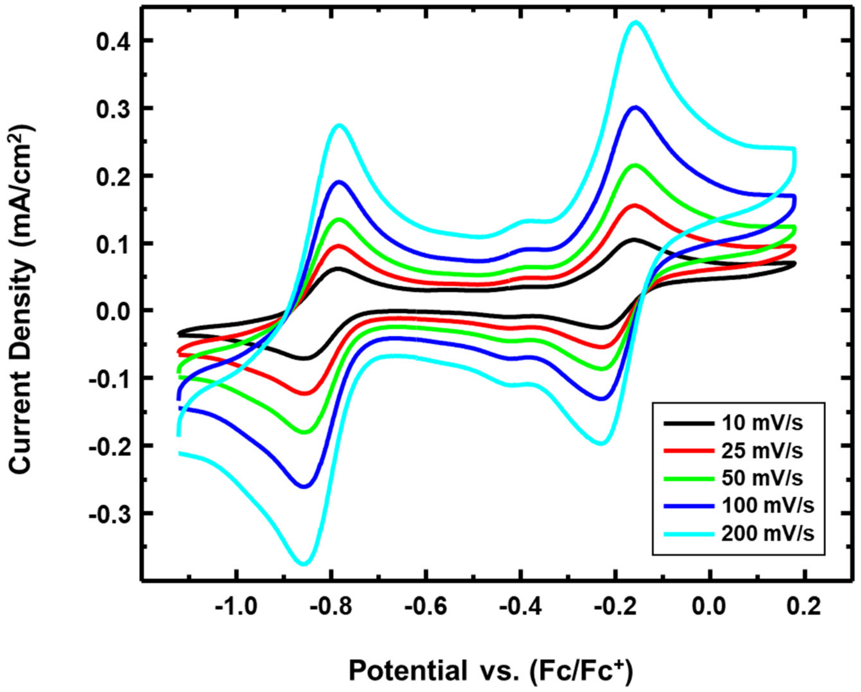

Figure S1. Variable scan rate CV for **3** in MeCN. Data were collected with 0.1 M ($^{n}Bu_4N)PF_6$ using a glassy carbon working electrode, Pt wire counter electrode, and Pt wire quasi-reference electrode. The CV scan shows two reversible redox features at $E_{1/2}$ = -0.82 V vs Fc/ Fc^+ (I_{pc}/I_{pa} = 0.981), -0.19 V vs Fc/ Fc^+ (I_{pc}/I_{pa} = 0.969). The quasi-reversible feature at $E_{1/2}$ = -0.40 V vs Fc/ Fc^+ is attributed to the presence of adventitious water.

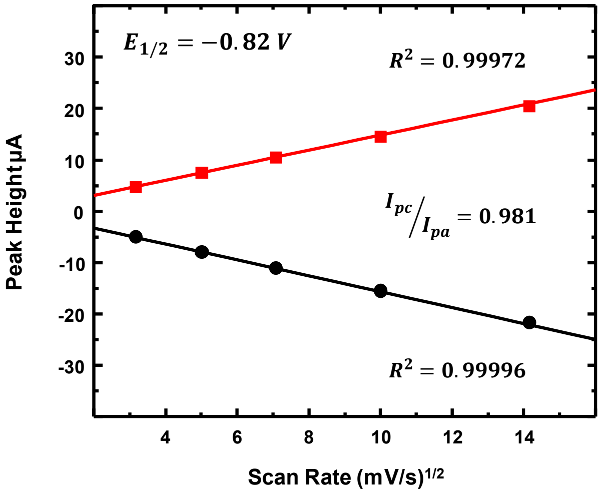

Figure S2. Red trace I_{pa} = (1.42·10^{-6})x + 3.86·10^{-6}, black trace I_{pc} = (-1.52·10^{-6})x – 2.15·10^{-6}. Above is a plot of the peak current density (lifted from CV data for the analyte at scan rates of 10, 25, 50, 100, and 200 mV/s) for the redox feature with an $E_{1/2}$ = -0.82 V (vs Fc/Fc^{+}). The linear relationship between the peak current density and the square root of the scan rate suggests that the redox active species are freely diffusing in the solution. Ratio of the cathodic and anodic current density (0.981), taken at 100 mV/s, reveals the reversibility of the redox feature (-0.82 V vs Fc/Fc^{+}) to be fully reversible.

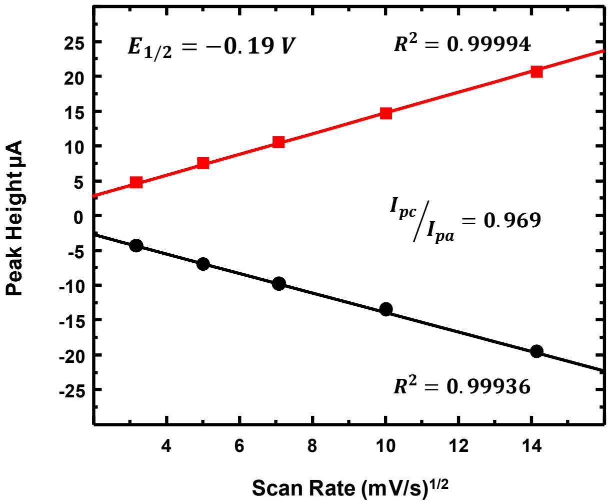

Figure S3. Red trace I_{pa} = (1.44·10^{-6})x + 3.45·10^{-6}, black trace I_{pc} = (-1.37·10^{-6})x – 6.78·10^{-8}. Above is a plot of the peak current density (lifted from CV data for the analyte at scan rates of 10, 25, 50, 100, and 200 mV/s) for the redox feature with an $E_{1/2}$ = -0.19 V (vs Fc/Fc^{+}). The linear relationship between the peak current density and the square root of the scan rate suggests that the redox active species are freely diffusing in the solution. Ratio of the cathodic and anodic current density (0.969), taken at 100 mV/s, reveals the reversibility of the redox feature (-0.19 V vs Fc/Fc^{+}) to be fully reversible.

Table S1. Crystallographic data for **$3(BF_4)_2$**.

Formula	$C_{40}H_{36}B_2F_8N_3PRuS_2$
FW (g mol^{-1})	928.50
crystal system	triclinic
space group	P-1
a (Å)	11.6409(12)
b (Å)	12.7721(13)
c (Å)	16.3060(16)
α (deg)	72.635(5)
β (deg)	71.514(5)
γ (deg)	74.580(5)
volume (Å^3)	2154.8(4)
Z	2
ρ_{calc} (g cm^{-3})	1.431
μ (mm^{-1})	0.564
F (000)	940
θ range (deg)	3.341/20.35
R(int)	0.0380
data/restraints/parameters	8775/0/517
GOF	1.044
R_1 [$I > 2\sigma(I)$][a]	0.0372
wR_2 (all data)[b]	0.0937
Ext. Coeff.	-
Largest Peak/Hole (e · Å^{-3})	0.443/-0.513
Temp (K)	150(2)

[a]$R_1 = \sum ||F_o| - |F_c|| / \sum|F_o|$ for reflections with $F_o^2 > 2\sigma(F_o^2)$

[b]$wR_2 = [\sum w(F_o^2 - F_c^2)^2 / \sum(F_o^2)^2]^{1/2}$ for all reflections.

Table S2. Comparison of select experimental and calculated bond distances and angles.

	**1** Exp	**1** Calc	**2** Exp	**2** Calc	**3** Exp	**3** Calc	**3(BF$_4$)** Exp	**3$^+$** Calc	**3(BF$_4$)$_2$** Exp	**3^{2+}** Calc (s)*	**3^{2+}** Calc (t)*
Ru-N	2.006(6)	2.051	2.039(4)	2.056	2.034(3)	2.058	2.022(2)	2.036	1.997(2)	2.013	2.010
	2.017(5)	2.046	2.097(4)	2.120	2.046(4)	2.063	2.015(2)	2.044	1.993(2)	2.022	2.009
Ru-S	2.330(2)	2.416	2.327(1)	2.384	2.330(1)	2.404	2.3502(8)	2.418	2.3740(8)	2.431	2.426
	2.338(2)	2.417	2.316(1)	2.386	2.323(1)	2.413	2.3416(7)	2.419	2.3767(8)	2.433	2.428
Ru-P	2.214(2)	2.246	2.317(1)	2.361	2.272(1)	2.325	2.3121(7)	2.386	2.354(1)	2.435	2.411
Ru-X†	-	-	1.77(4)	1.771	2.136(3)	2.130	2.119(2)	2.099	2.084(3)	2.106	2.147
C-N	1.39(1)	1.408	1.385(5)	1.389	1.378(5)	1.401	1.376(3)	1.372	1.348(3)	1.345	1.403
(backbone)	1.39(1)	1.405	1.465(6)	1.468	1.378(6)	1.400	1.376(3)	1.375	1.341(3)	1.348	1.406
C-N	1.39(1)	1.380	1.374(6)	1.364	1.390(6)	1.361	1.393(3)	1.386	1.415(3)	1.405	1.383
(flanking)	1.40(1)	1.383	1.452(6)	1.446	1.391(7)	1.366	1.398(3)	1.388	1.418(4)	1.404	1.381
NC-CN	1.45(1)	1.439	1.418(7)	1.433	1.442(6)	1.439	1.438(4)	1.453	1.460(4)	1.473	1.434
N-Ru-N	79.8(2)	79.4	80.2(1)	80.2	80.9(1)	80.9	80.33(8)	80.4	80.1(1)	80.1	81.7
S-Ru-S	107.84(8)	110.3	110.23(4)	110.6	109.49(4)	111.9	111.61(3)	111.9	112.00(3)	111.7	112.8
N-Ru-S	84.1(2)	83.7	83.9(1)	83.7	84.6(1)	84.1	84.10(6)	83.5	84.41(7)	84.5	83.1
	84.1(2)	83.5	85.5(1)	85.5	84.7(1)	82.8	83.85(6)	84.2	83.51(7)	83.6	82.2
	159.1(2)	159.7	158.5(1)	156.8	164.6(1)	163.3	163.84(6)	164.5	163.59(7)	164.7	163.7
	159.3(2)	159.0	165.7(1)	165.5	165.1(1)	164.5	164.40(6)	163.9	164.39(7)	163.7	164.6
P-Ru-X†	-	-	178(1)	177.6	175.7(1)	173.3	179.65(6)	177.3	175.60(7)	177.4	179.1

*s = singlet, t = triplet; †X = H or MeCN

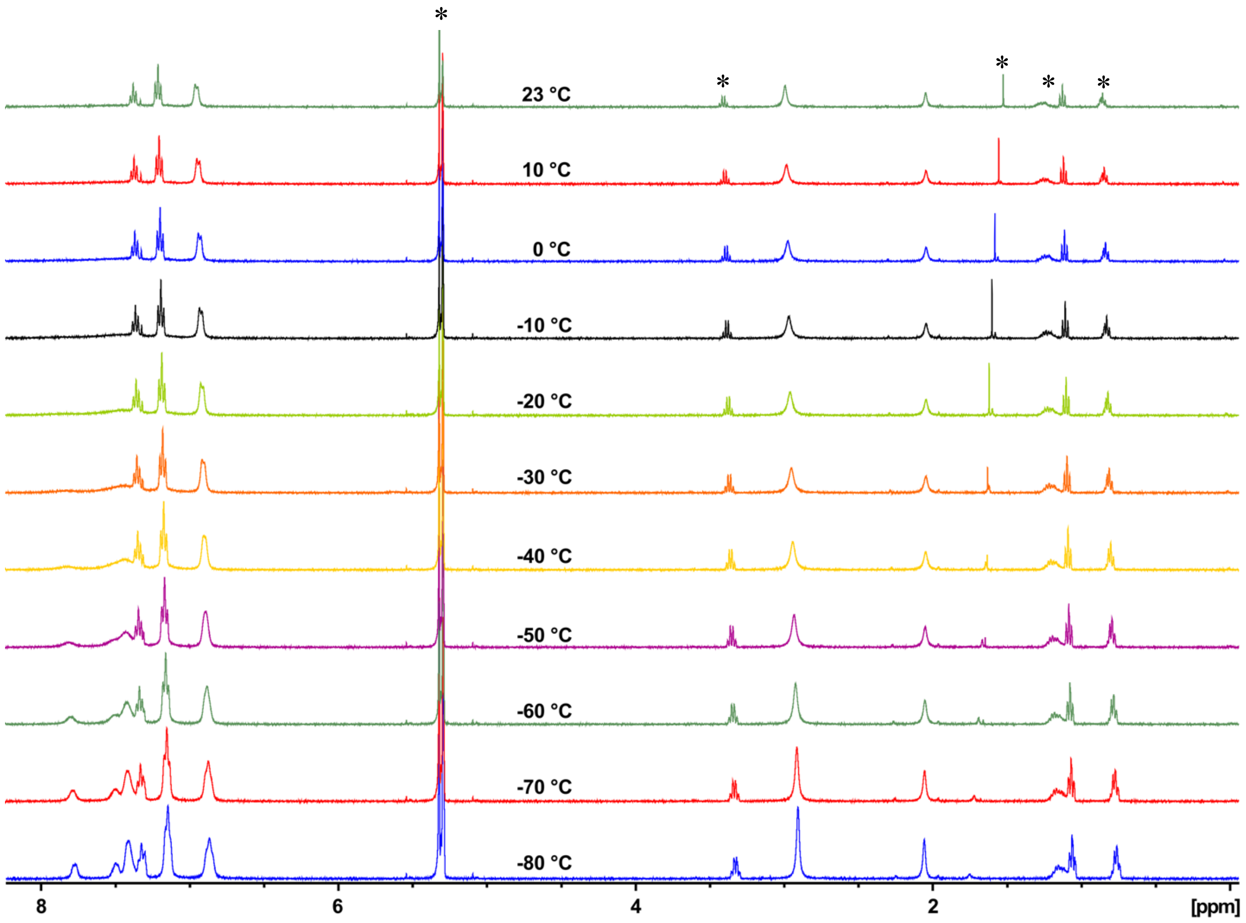

Figure S4. Variable temperature ^{1}H NMR spectrum of **$3(BF_4)_2$** in CD_2Cl_2. The asterisks indicate features assigned to residual solvent impurities.

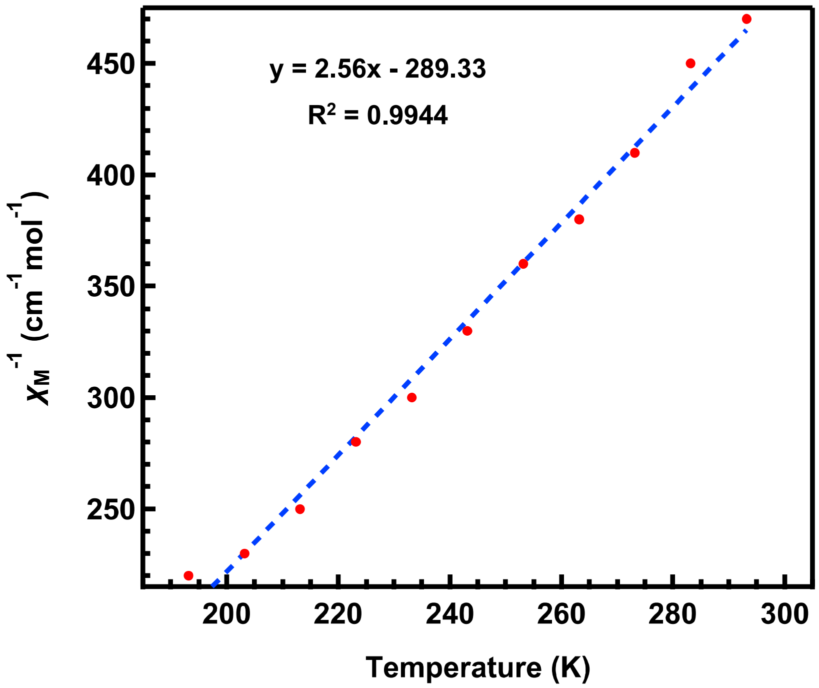

Figure S5. Curie-Weiss plot of VT Evans method experiment for **3**(**BF_4**) (3.6 μM in DCM with a DCM capillary insert). Collected using a DRX Bruker 400 MHz NMR instrument. Temp: 193 K (-80 °C) to 293 K (20 °C) at 10 K increments with a protio/deuterated CH_2Cl_2 capillary insert and at a range of 2 - 10 μM.

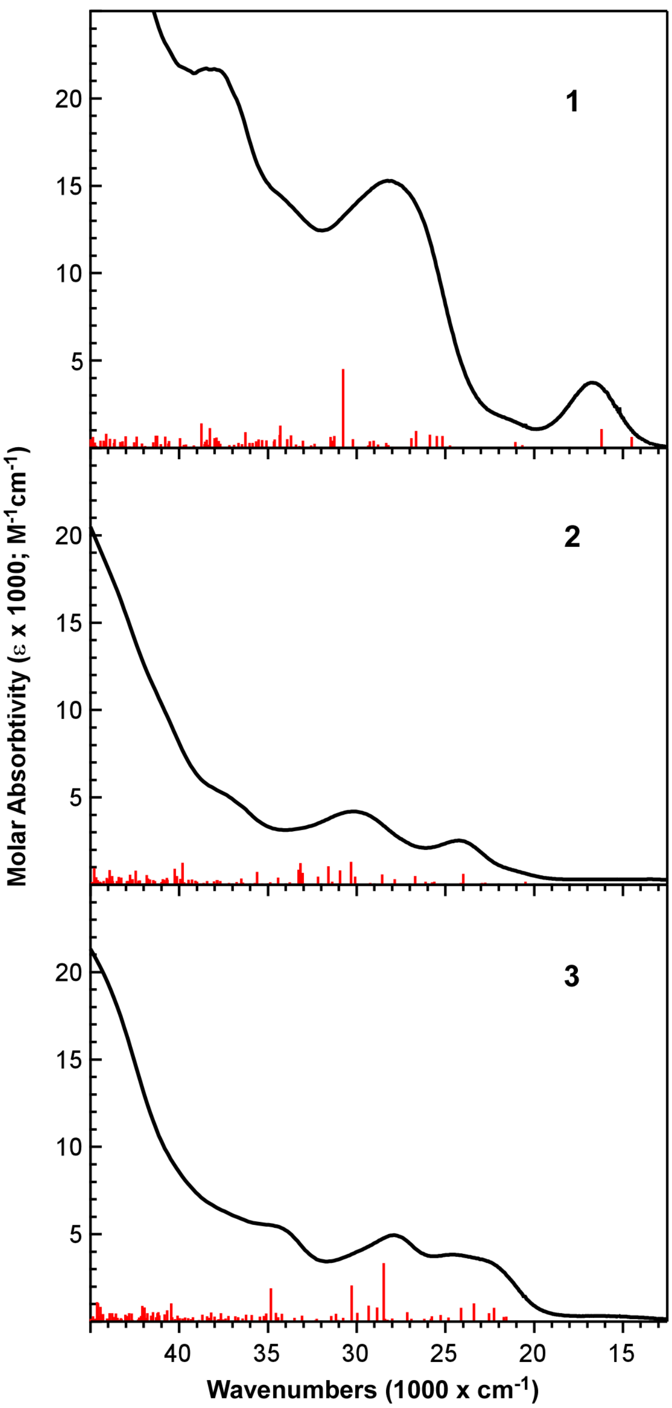

Figure S6. Comparison of UV-vis data collected on isolated samples of **1** in thf, **2** in thf, and **3** in MeCN to calculated transitions from TDDFT. Calculated oscillator strengths for the transitions have been multiplied by a factor of 3.0 x 10^4 to bring them on scale with the experimental results.

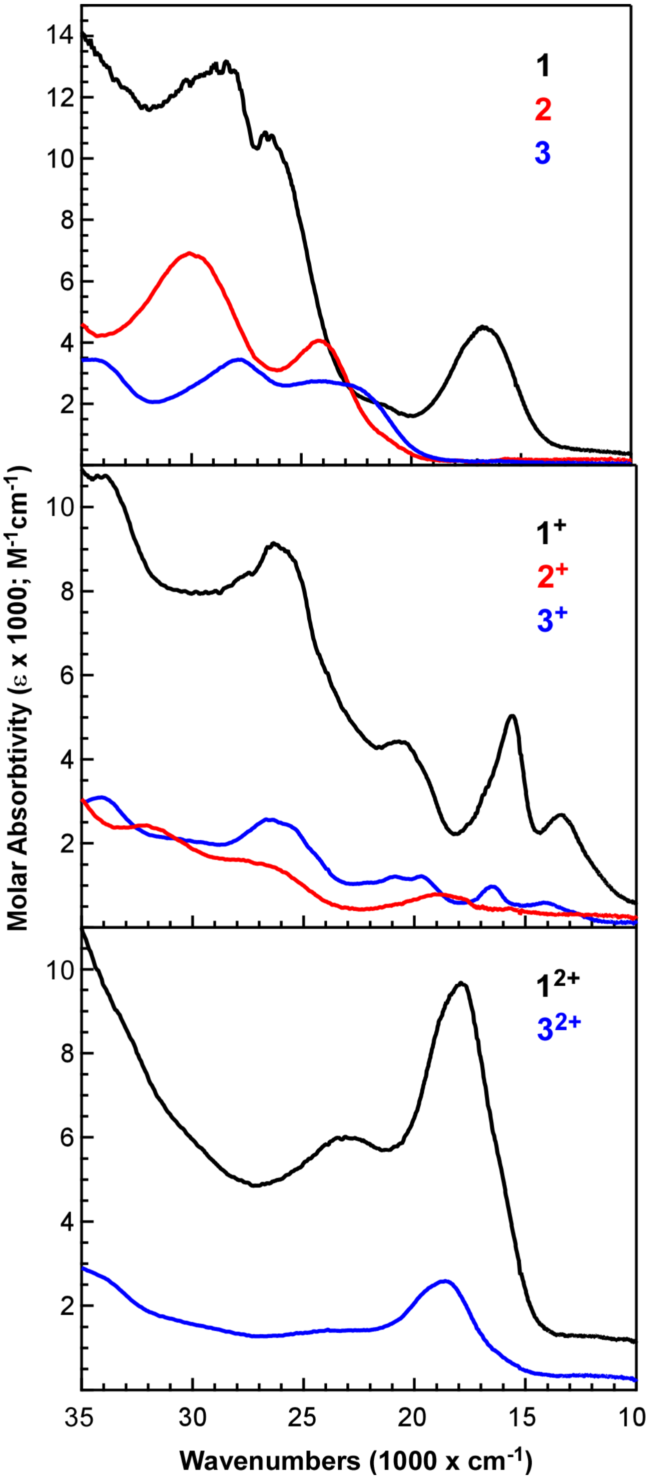

Figure S7. Comparison of the UV-vis SEC spectra of neutral and oxidized samples of **1** (black), **2** (red), and **3** (blue).

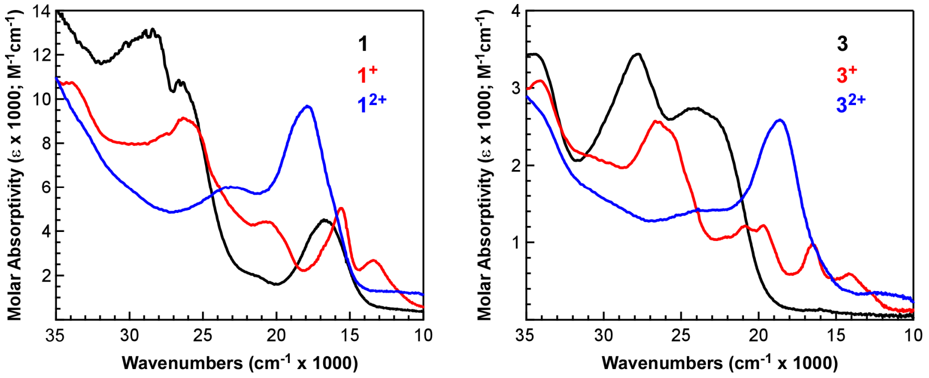

Figure S8. Comparison of the UV-vis SEC spectra of neutral and oxidized samples of **1** (left) and **3** (right).

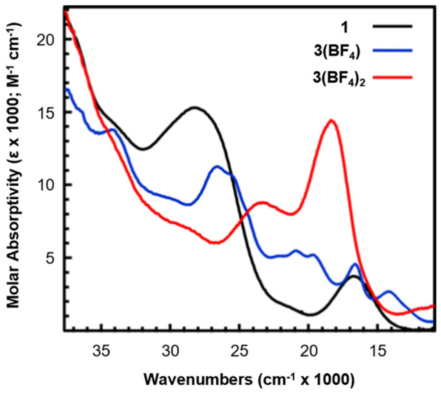

Figure S9. Comparison of UV-vis spectra for **1** (black), **$3(BF_4)$** (blue), and **$3(BF_4)_2$** (red). Data were collected on 50 μM solutions in CH_2Cl_2.

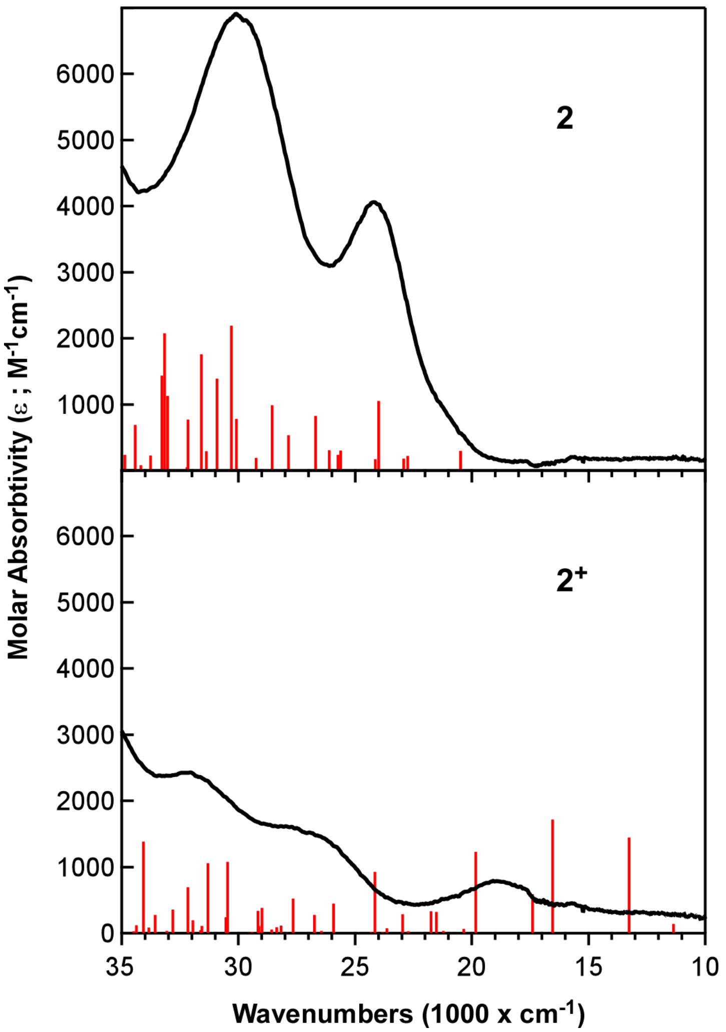

Figure S10. Comparison of the UV-vis SEC spectra of **2** and $\mathbf{2}^{+}$ to calculated transitions from TDDFT. The plot of the doubly oxidized spectrum on the left shows TDDFT transitions for both the calculated singlet (red) and triplet (blue) configurations. The plots on the right compare the calculated singlet and triplet transitions separately for comparison. Calculated oscillator strengths for the transitions have been multiplied by a factor of 5.0 x 10^4 to bring them on scale with the experimental results.

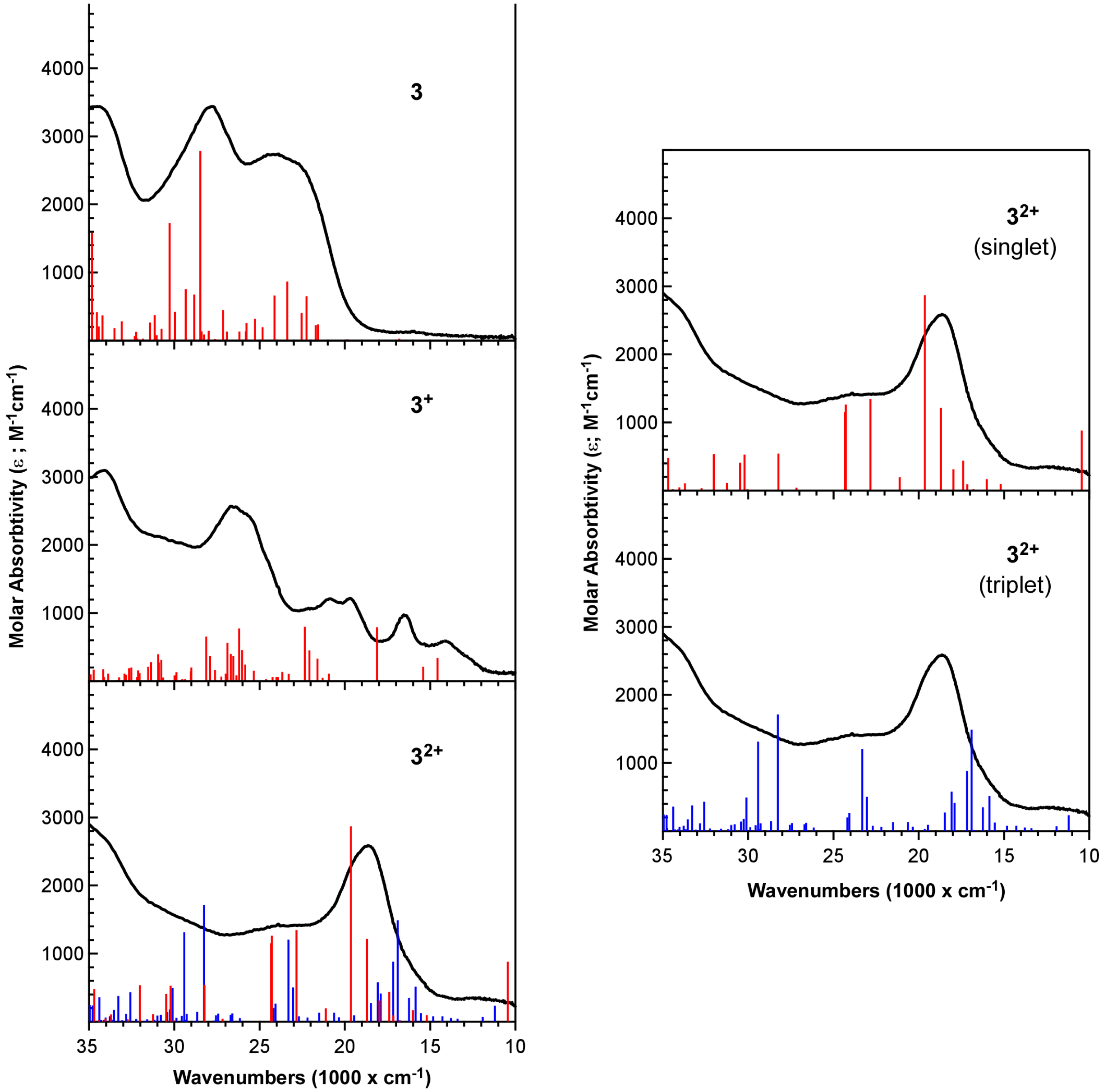

Figure S11. Comparison of the UV-vis SEC spectra of **3**, **3**$^{+}$, and **3**$^{2+}$ to calculated transitions from TDDFT. The plot of the doubly oxidized spectrum on the left shows TDDFT transitions for both the calculated singlet (red) and triplet (blue) configurations. The plots on the right compare the calculated singlet and triplet transitions separately for comparison. Calculated oscillator strengths for the transitions have been multiplied by a factor of 2.5 x 10^4 to bring them on scale with the experimental results.

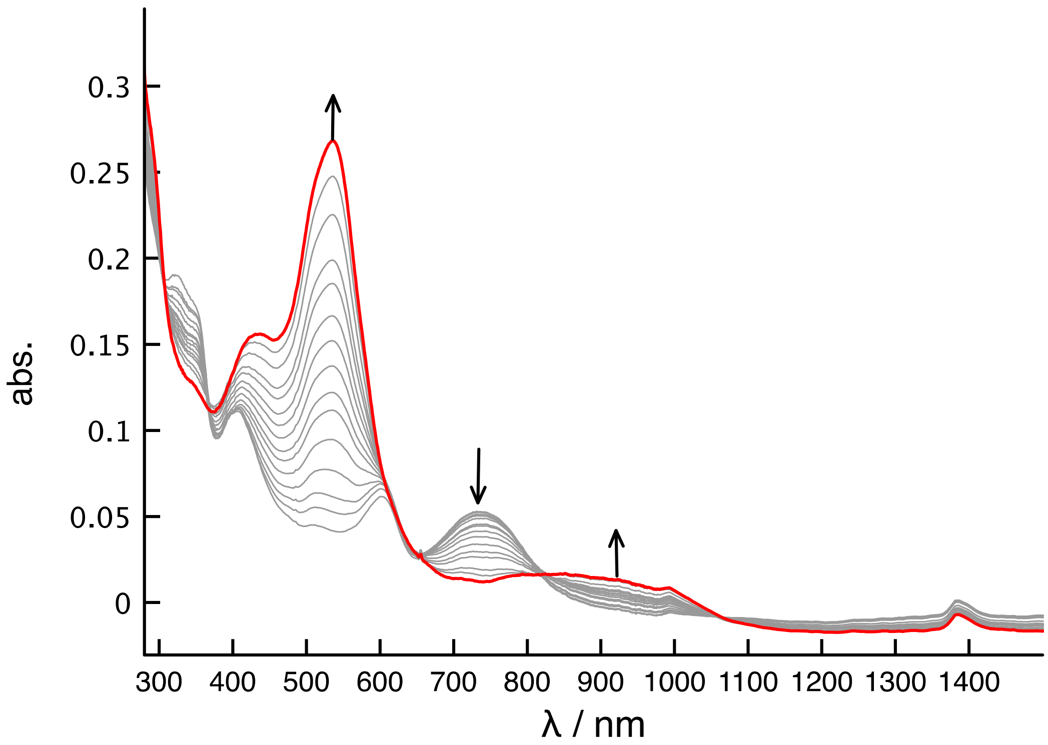

Figure S12. UV-vis-NIR SEC spectra of **3**$^{+}$ being oxidized to **3**$^{2+}$ in MeCN.

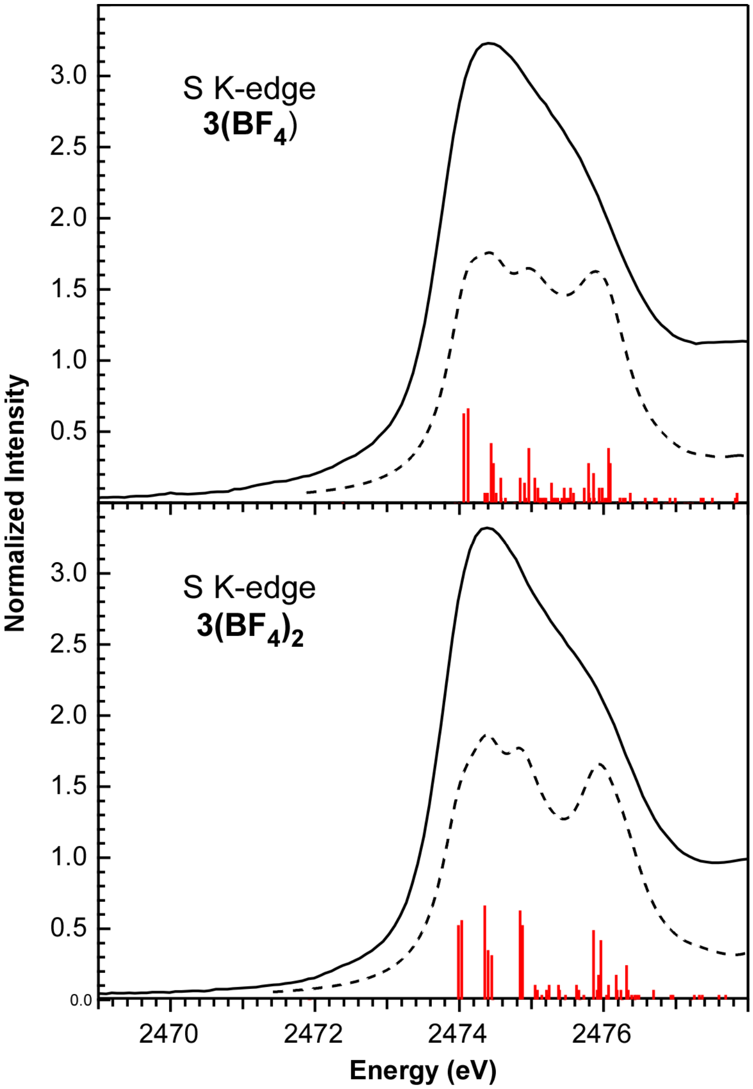

Figure S13. Comparison of experimental and simulated S K-edge XAS data for **1** and **2**. The experimental spectra (solid lines), simulated spectra (dashed lines), and calculated transitions (red bars) are shown. Calculated oscillator strengths were multiplied by a factor of 350 to bring them on scale with the experimental data. As described previously,[1-4] an energy shift of +57.0 eV (S K) was applied to the calculated spectra so that relative differences in calculated and experimental peak positions could be compared.

CASSCF/CASPT2 Calculations

Table S3. CASPT2 singlet and triplet energies (kcal/mol) of **3^{2+}** on DFT-optimized singlet and triplet structures with a (2e,2o) active space.

Geometry	Spin-State (2e,2o)	
	**Singlet**	**Triplet**
**Singlet**	1.94	22.36
**Triplet**	0.00	**17.52**

Table S4. CASPT2 singlet and triplet energies (kcal/mol) of **3^{2+}** on DFT-optimized singlet and triplet structures with a (6e,6o) active space.

Geometry	Spin-State (6e,6o)	
	**Singlet**	**Triplet**
**Singlet**	**2.84**	26.12
**Triplet**	0.00	**21.39**

Bonding and Radical Character

The radical character is assessed by use of the effective bond order (EBO). EBOs are calculated as the difference between the natural orbital occupation numbers (NOON) from one set of bonding and antibonding pairs attained from CASSCF.

$$EBO = \frac{NOON_{bonding} - NOON_{antibonding}}{2}$$

From the EBO, the percentage of radical character (% rad) can be defined as

$$\%rad = (1 - EBO) * 100$$

For example, a closed shell singlet would have an EBO of 1 with 0% radical character. A triplet would give an EBO of 0 with 100% radical character. Anything with multiconfigurational character will fall somewhere in between.

Table S5. CASPT2 singlet and triplet Effective Bond Orders (EBOs) of $\mathbf{3}^{2+}$ on DFT-optimized singlet and triplet structures with a (2e,2o) active space.

**Geometry**	**Spin-State (2e,2o)**	
	**Singlet**	**Triplet**
**Singlet**	1.00	0.00
**Triplet**	0.63	0.00

Table S6. CASPT2 singlet and triplet %rad character of $\mathbf{3}^{2+}$ on DFT-optimized singlet and triplet structures with a (2e,2o) active space.

**Geometry**	**Spin-State (2e,2o)**	
	**Singlet**	**Triplet**
**Singlet**	0%	100%
**Triplet**	36%	100%

Table S7. CASPT2 singlet and triplet Effective Bond Orders (EBOs) of $\mathbf{3^{2+}}$ on DFT-optimized singlet and triplet structures with a (6e,6o) active space.

**Geometry**	**Spin-State (6e,6o)**	
	**Singlet**	**Triplet**
**Singlet**	0.93	0.00
**Triplet**	0.79	0.00

Table S8. CASPT2 singlet and triplet %rad character of $\mathbf{3^{2+}}$ on DFT-optimized singlet and triplet structures with a (6e,6o) active space.

**Geometry**	**Spin-State (6e,6o)**	
	**Singlet**	**Triplet**
**Singlet**	7%	100%
**Triplet**	21%	100%

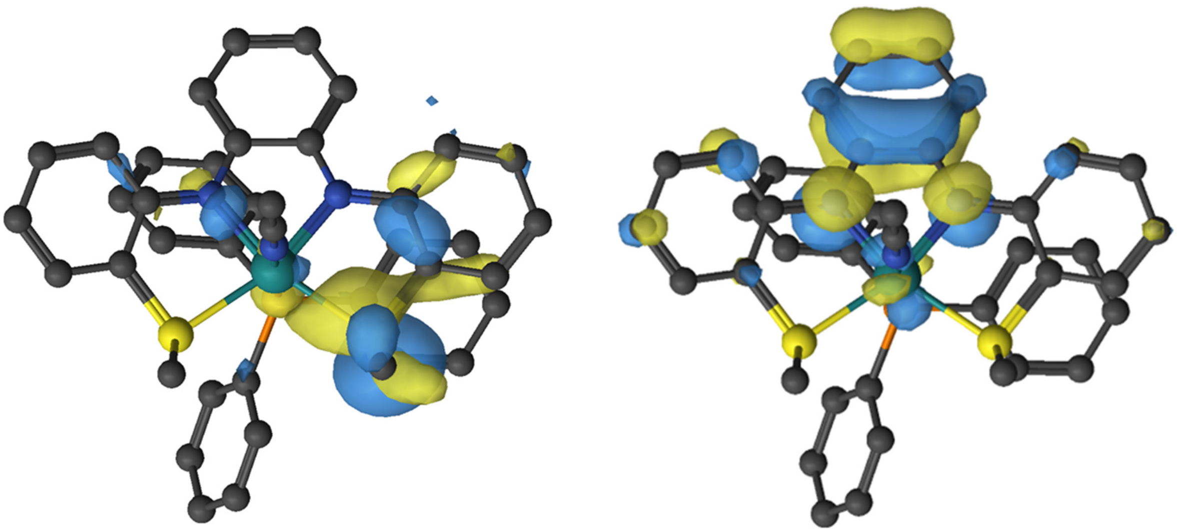

(2.00) (0.00)

Figure S14. CASSCF active natural orbitals and the corresponding occupation numbers using a (2e,2o) active space for a singlet spin-state on the singlet DFT geometry of **3**$^{2+}$.

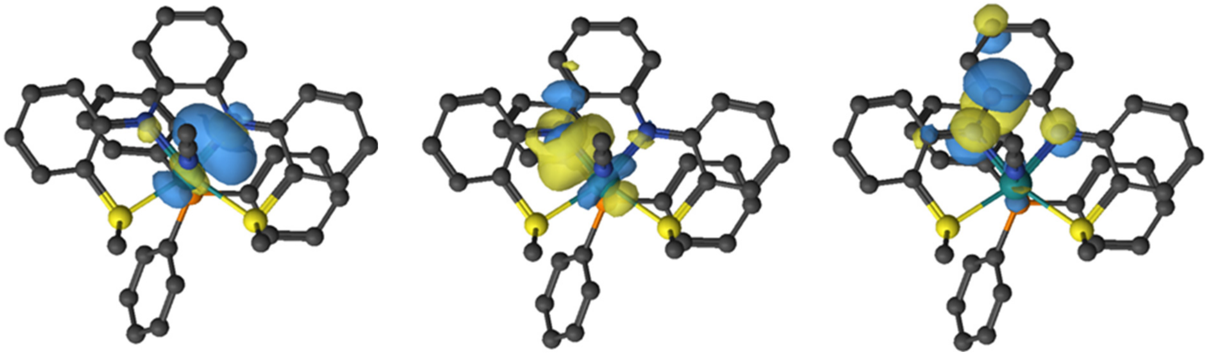

(0.01) (0.02) (0.07)

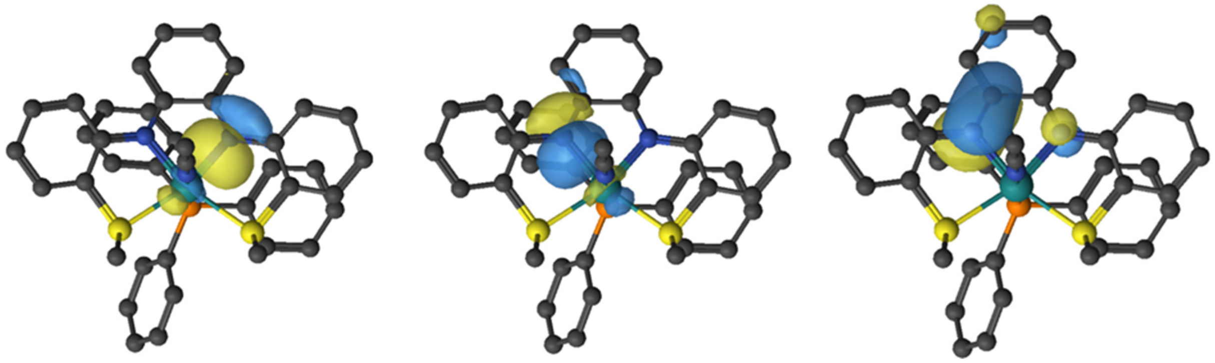

(1.97) (1.98) (1.93)

Figure S15. CASSCF active natural orbitals and the corresponding occupation numbers using a (6e,6o) active space for a singlet spin-state on the singlet DFT geometry of **3**$^{2+}$.

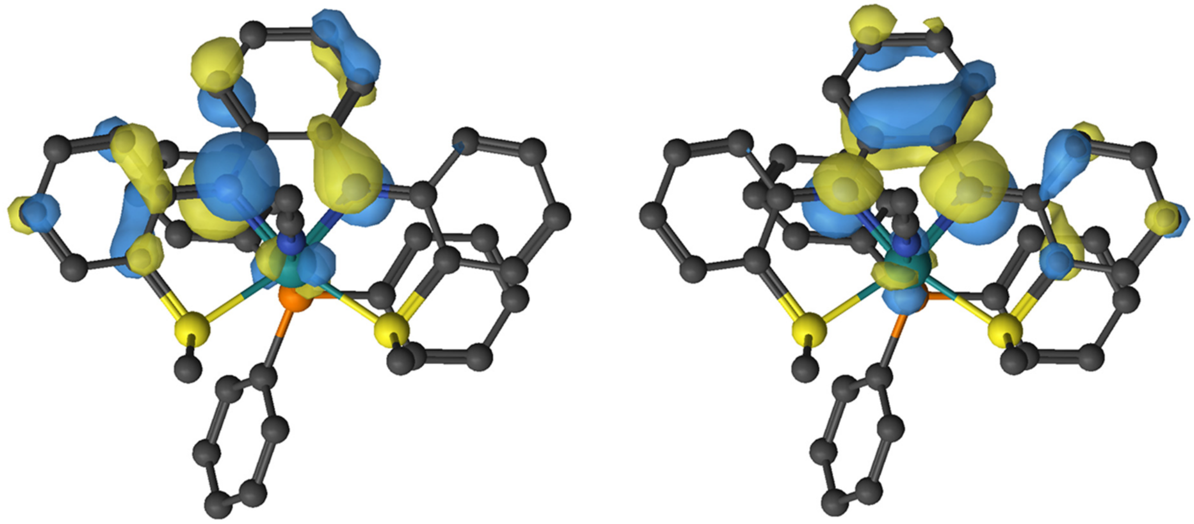

(1.00) **(1.00)**

Figure S16. CASSCF active natural orbitals and the corresponding occupation numbers using a (2e,2o) active space for a triplet spin-state on the singlet DFT geometry of $\mathbf{3}^{2+}$.

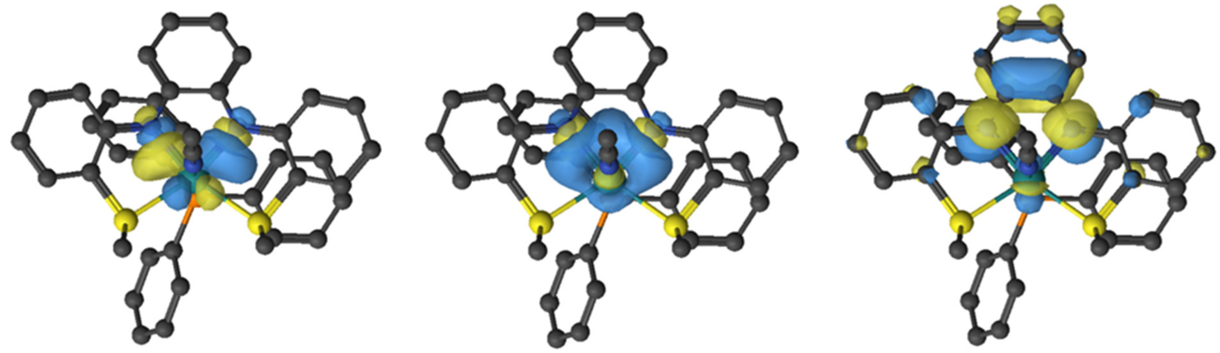

(0.01) **(0.01)** **(1.00)**

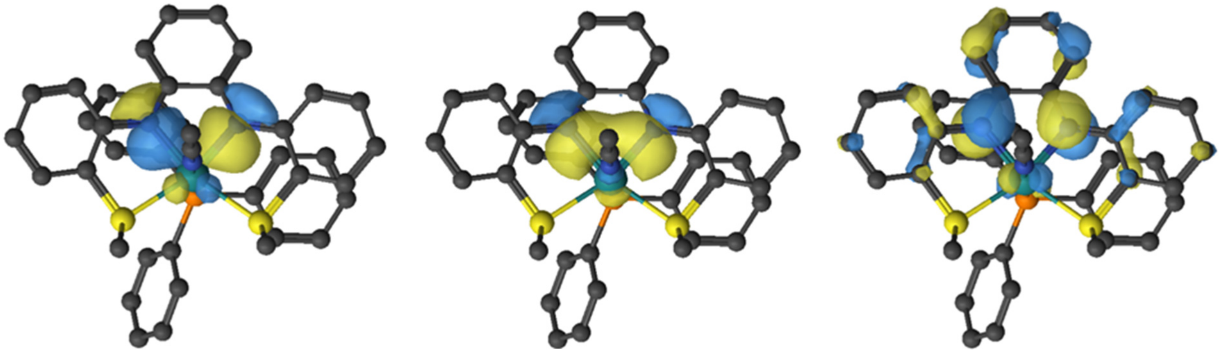

(1.99) **(1.99)** **(1.00)**

Figure S17. CASSCF active natural orbitals and the corresponding occupation numbers using a (6e,6o) active space for a triplet spin-state on the singlet DFT geometry of $\mathbf{3}^{2+}$.

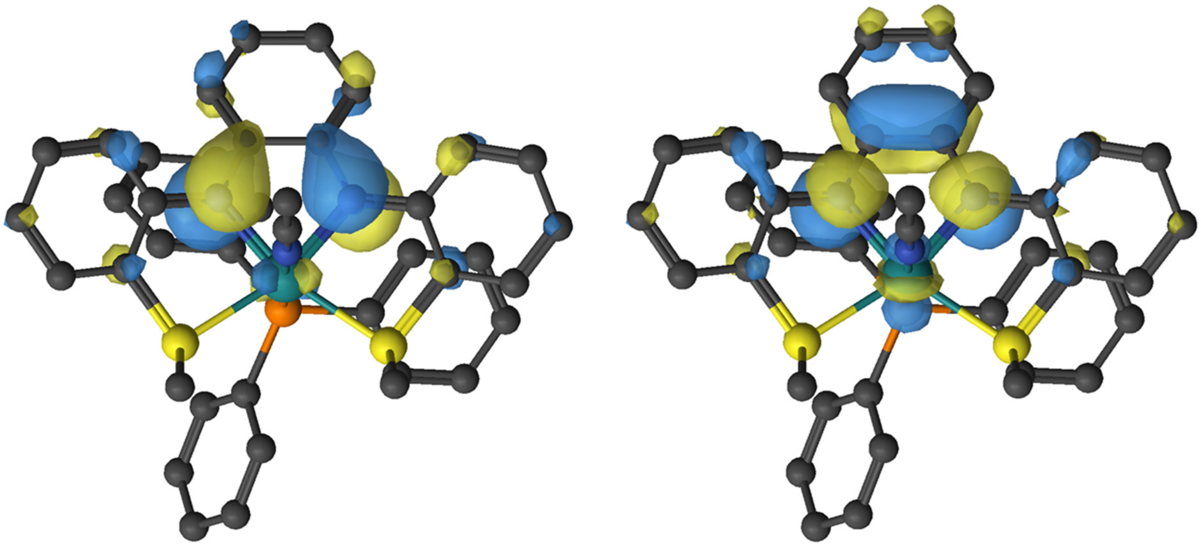

(1.64) (0.36)

Figure S18. CASSCF active natural orbitals and the corresponding occupation numbers using a (2e,2o) active space for a singlet spin-state on the triplet DFT geometry of $\mathbf{3}^{2+}$.

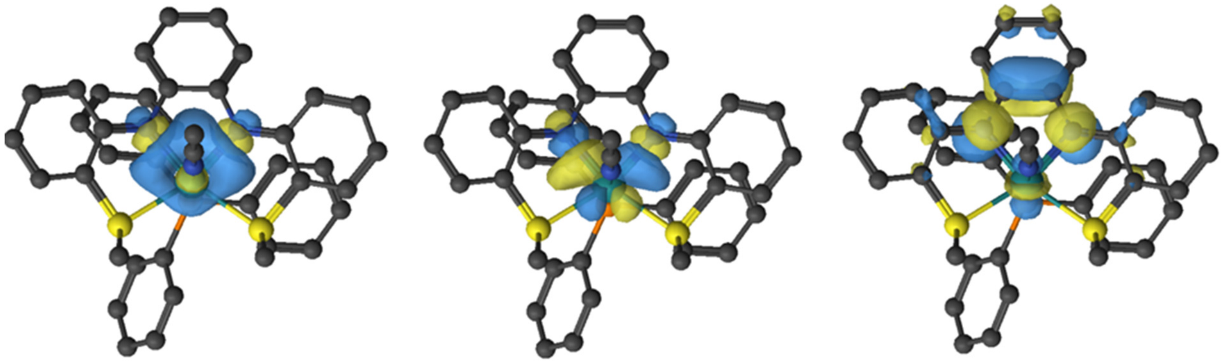

(0.02) (0.02) (0.21)

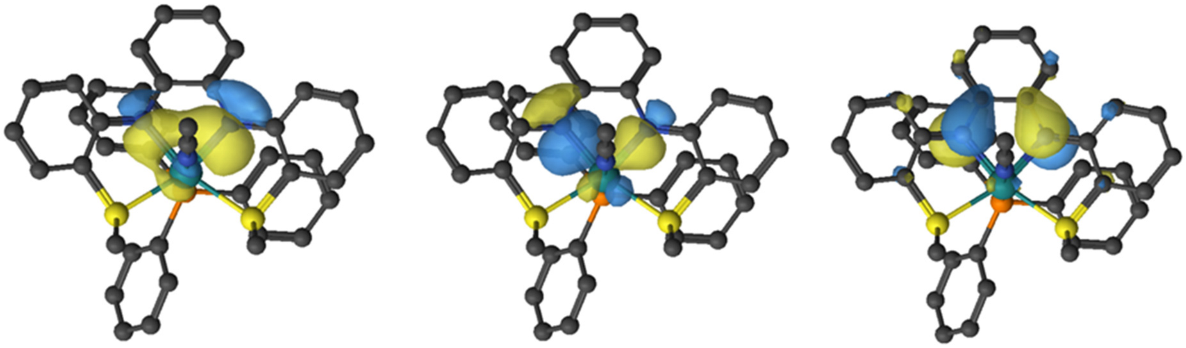

(1.98) (1.98) (1.79)

Figure S19. CASSCF active natural orbitals and the corresponding occupation numbers using a (6e,6o) active space for a singlet spin-state on the triplet DFT geometry of $\mathbf{3}^{2+}$.

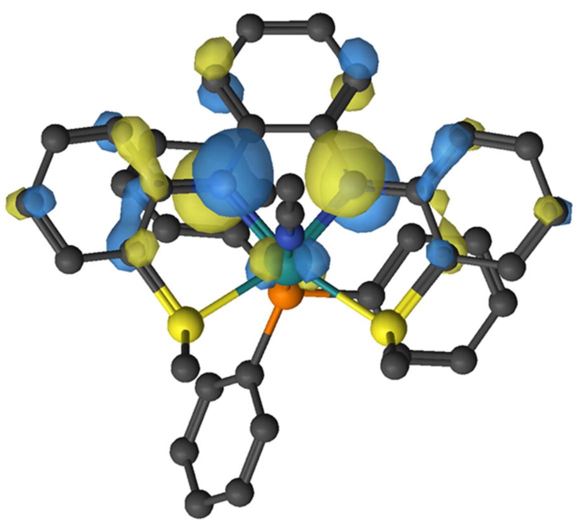

(1.00) **(1.00)**

Figure S20. CASSCF active natural orbitals and the corresponding occupation numbers using a (2e,2o) active space for a triplet spin-state on the triplet DFT geometry of $\mathbf{3}^{2+}$.

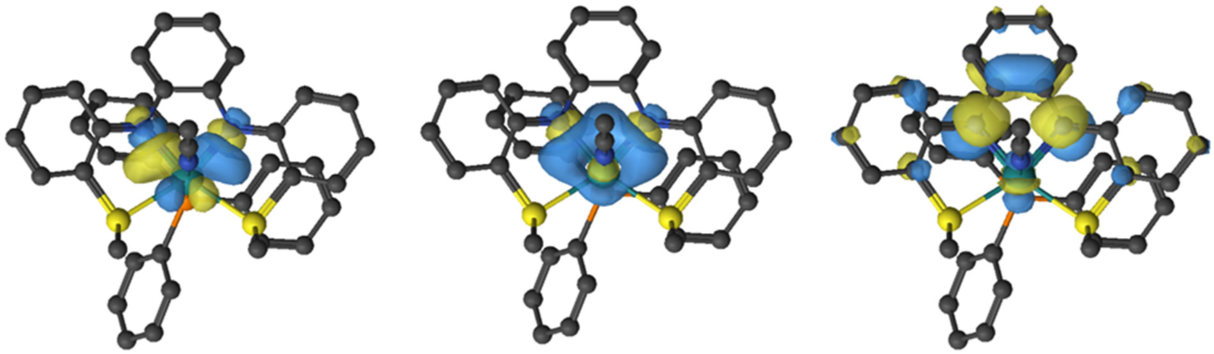

(0.02) **(0.01)** **(1.00)**

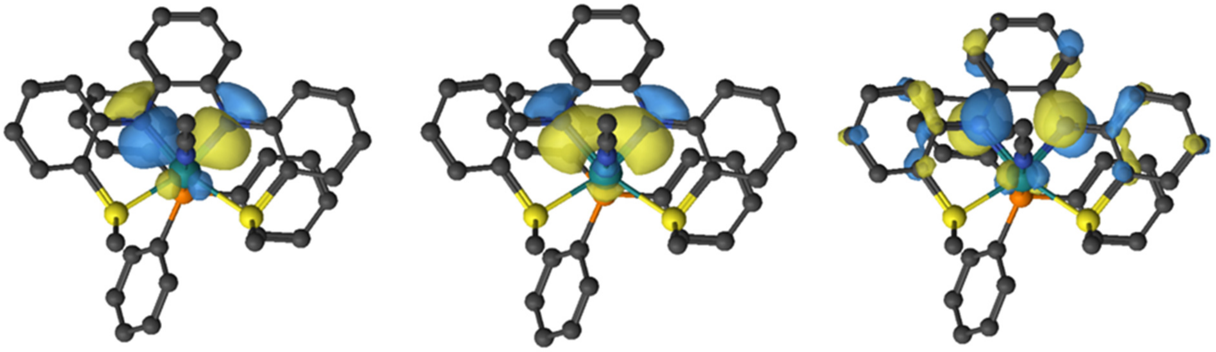

(1.98) **(1.99)** **(1.00)**

Figure S21. CASSCF active natural orbitals and the corresponding occupation numbers using a (6e,6o) active space for a triplet spin-state on the triplet DFT geometry of $\mathbf{3}^{2+}$.

Supporting Information References

(1) Donahue, C. M.; Lezama Pacheco, J. S.; Keith, J. M.; Daly, S. R. Sulfur K-edge X-ray Absorption Spectroscopy and Time-Dependent Density Functional Theory of Arsenic Dithiocarbamates. *Dalton Trans.* **2014**, *43*, 9189-9201.

(2) Olson, A. C.; Keith, J. M.; Batista, E. R.; Boland, K. S.; Daly, S. R.; Kozimor, S. A.; MacInnes, M. M.; Martin, R. L.; Scott, B. L. Using Solution- and Solid-State S K-edge X-ray Absorption Spectroscopy with Density Functional Theory to Evaluate M-S bonding for MS_4^{2-} (M = Cr, Mo, W) Dianions. *Dalton Trans.* **2014**, *43*, 17283-17295.

(3) Donahue, C. M.; McCollom, S. P.; Forrest, C. M.; Blake, A. V.; Bellott, B. J.; Keith, J. M.; Daly, S. R. Impact of Coordination Geometry, Bite Angle, and Trans Influence on Metal-Ligand Covalency in Phenyl-Substituted Phosphine Complexes of Ni and Pd. *Inorg. Chem.* **2015**, *54*, 5646-5659.

(4) Lee, K.; Wei, H.; Blake, A. V.; Donahue, C. M.; Keith, J. M.; Daly, S. R. Ligand K-edge XAS, DFT, and TDDFT Analysis of Pincer Linker Variations in Rh(I) PNP Complexes: Reactivity Insights from Electronic Structure. *Dalton Trans.* **2016**, *45*, 9774-9785.
